# Precise tuning in platinum-nickel/nickel sulfide interface nanowires for synergistic hydrogen evolution catalysis

**DOI:** 10.1038/ncomms14580

**Published:** 2017-02-27

**Authors:** Pengtang Wang, Xu Zhang, Jin Zhang, Sheng Wan, Shaojun Guo, Gang Lu, Jianlin Yao, Xiaoqing Huang

**Affiliations:** 1 College of Chemistry, Chemical Engineering and Materials Science, Soochow University, Jiangsu 215123, China; 2 Department of Physics and Astronomy, California State University, Northridge, California 91330, USA; 3 Department of Materials Science and Engineering, College of Engineering, Peking University, Beijing 100871, China; 4 BIC-ESAT, College of Engineering, Peking University, Beijing 100871, China; 5 Department of Energy and Resources Engineering, College of Engineering, Peking University, Beijing 100871, China

## Abstract

Comprising abundant interfaces, multicomponent heterostructures can integrate distinct building blocks into single entities and yield exceptional functionalities enabled by the synergistic components. Here we report an efficient approach to construct one-dimensional metal/sulfide heterostructures by directly sulfuring highly composition-segregated platinum-nickel nanowires. The heterostructures possess a high density of interfaces between platinum-nickel and nickel sulfide components, which cooperate synergistically towards alkaline hydrogen evolution reaction. The platinum-nickel/nickel sulfide heterostructures can deliver a current density of 37.2 mA cm^−2^ at an overpotential of 70 mV, which is 9.7 times higher than that of commercial Pt/C. The heterostructures also offer enhanced stability revealed by long-term chronopotentiometry measurements. The present work highlights a potentially powerful interface-engineering strategy for designing multicomponent heterostructures with advanced performance in hydrogen evolution reaction and beyond.

## Abstract (web summary)

Multicomponent, nanoscale heterostructures may exhibit notable catalytic properties imparted by the various building blocks. Here, the authors fabricate metal/sulfide heterostructures via direct sulfurization of segregated platinum-nickel nanowires, and assess their hydrogen evolution performance.

Hydrogen (H_2_), as a clean and renewable energy resource, has been considered as a promising alternative to replace the diminishing fossil fuel^1^,^2^,^3^,^4^. The grand challenge leading to extensive use of hydrogen energy system is to produce H_2_ in an efficient and cost-effective manner. Electrochemical water splitting by combining hydrogen evolution reaction (HER) and oxygen evolution reaction is attracting much attention due to its inherent advantages including accessible reactants, stable output, feasibility of large-scale production and highly pure product^5^,^6^,^7^,^8^,^9^,^10^. In general, HER is believed to consist of two pathways: Volmer/Tafel pathway or Volmer/Heyrovsky pathway^11^,^12^,^13^. However, the Heyrovsky and the Volmer steps have different expressions in acidic or basic media^11^,^12^,^13^. The reacting species are H_2_O/OH^−^ in the base solution and H^+^ in the acid solution; thus, the cleavage of HO–H bond in H_2_O is crucial for alkaline HER^13^,^14^. To date, platinum (Pt) is generally considered as one of the best catalysts towards HER, particularly in acid media^15^,^16^,^17^,^18^. However, the HER kinetics of Pt is much slower in the alkaline condition, resulting in the HER activity that is approximately two to three orders of magnitude lower than in the acidic media^19^,^20^. This may be attributed to the fact that although Pt is conducive to the adsorption of reactive hydrogen intermediates (H_ads_) and their combination into H_2_ molecules, it is unfortunately not efficient in splitting water into H_ads_ in the alkaline solution^13^,^21^,^22^. The slow kinetics in the alkaline solution leads to low efficiencies in both water-alkali and chlor-alkali electrolysers. Therefore, introducing ‘promoters' with the function to cleave HO–H bonds could open new opportunities to further enhance alkaline HER electrocatalysis^13^,^14^,^23^. However, which ‘promoter' can work well with Pt to yield more efficient and durable HER catalysts remains an open question.

Owing to intrinsic difference in the chemical reactivity of the metallic components, bimetallic nanocrystals with high composition segregation are exploited to develop unusual nanostructures with desirable functionalities^24^,^25^,^26^,^27^,^28^,^29^,^30^. They can create interior vacancies or open skeletons such as nanocages and nanoframes by readily sacrificing the relatively active metals^27^,^28^,^29^,^30^. Herein, we take the advantage of the highly composition-segregated Pt-Ni nanowires (NWs), to create a class of Pt_3_Ni/NiS heterostructures via a simple yet efficient sulfuration process. The Pt_3_Ni/NiS heterostructures with a high density of interfaces between NiS and Pt_3_Ni display excellent HER activity in both acidic and alkaline conditions. Specially, the optimized Pt_3_Ni_2_ NWs-S/C yield the highest activity in the alkaline condition with a current density of 37.2 mA cm^−2^ at an overpotential of 70 mV, which is 9.7 times higher than that of the commercial Pt/C, representing the best electrocatalysts towards alkaline HER, to the best of our knowledge. The density functional theory (DFT) calculations reveal that the synergy between NiS and Pt_3_Ni components can substantially enhance the HER activity in the alkaline solution with NiS promoting water dissociation, whereas Pt_3_Ni efficiently convert H^+^ to H_2_. Moreover, these heterostructures also exhibit enhanced HER stability with limited activity decay after a long-term chronopotentiometry run. The unprecedented catalytic performance offered by the novel Pt_3_Ni/NiS heterostructures highlights the importance of interfacial engineering in multicomponent electrocatalysts.

## Results

### Synthesis and characterization of PtNi/NiS NWs

A two-step procedure was used to make the unusual Pt_3_Ni/NiS heterostructures through first making the highly composition-segregated Pt-Ni NWs^31^ and then reacting them with sulfur in oleylamine (OAm) at high temperature. Supplementary Fig. 1 shows the transmission electron microscopy (TEM) images of the pristine Pt-Ni NWs before sulfuration, where abundant of NWs was observed. Through tuning the amounts of Ni(acac)_2_ and Pt(acac)_2_ precursors, the molar ratio of Pt to Ni in Pt-Ni NWs can be easily tuned from 3:1 to 3:2, 3:3 and 3:4, determined by scanning electron microscopy coupled with energy-dispersive X-ray spectroscopy (SEM–EDS) (Supplementary Fig. 1). The unique part for the composition tuning in Pt-Ni NWs is that with the Ni increasing, the Pt-Ni NWs become coarser and the composition-segregation features are more obvious, confirmed by both TEM images and powder X-ray diffraction (XRD) patterns (Supplementary Fig. 2).

**Figure 1:**
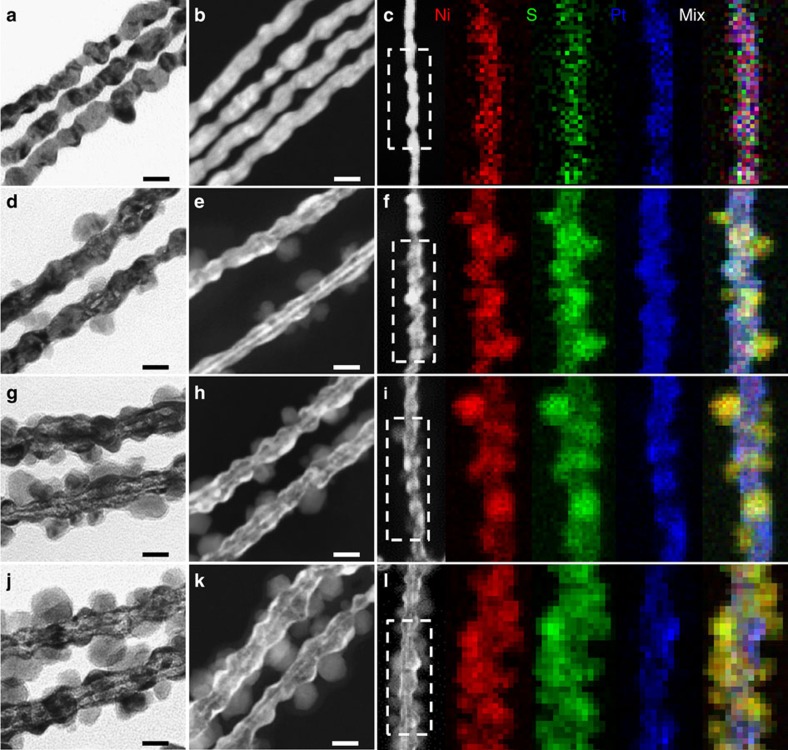
Morphology characterization and element distribution of Pt-Ni NWs-S. Representative (**a**,**d**,**g**,**j**) high-magnification TEM images, (**b**,**e**,**h**,**k**) HAADF–STEM images and (**c**,**f**,**i**,**l**) HAADF–STEM images, and corresponding EDS elemental mappings (Ni in red, S in green and Pt in blue) of (**a**–**c**) Pt_3_Ni_1_ NWs-S, (**d**–**f**) Pt_3_Ni_2_ NWs-S, (**g**–**i**) Pt_3_Ni_3_ NWs-S and (**j**–**l**) Pt_3_Ni_4_ NWs-S. Scale bars, 20 nm (**a**,**b**,**d**,**e**,**g**,**h**,**j**,**k**).

The highly composition-segregated Pt-Ni NWs were then subjected to sulfuration at high temperature (see details in Methods), as schematically illustrated in Supplementary Fig. 3. Figure 1 and Supplementary Fig. 4 show TEM and high-angle annular dark-field scanning TEM (HAADF–STEM) images of Pt_3_Ni_1_ NWs-S, Pt_3_Ni_2_ NWs-S, Pt_3_Ni_3_ NWs-S and Pt_3_Ni_4_ NWs-S by the sulfuration of Pt_3_Ni_1_ NWs, Pt_3_Ni_2_ NWs, Pt_3_Ni_3_ NWs and Pt_3_Ni_4_ NWs, respectively. After the sulfuration, all the Pt-Ni NWs-S can nicely maintain their original one-dimensional structure without exception, indicating sulfuration does not destroy the structure of Pt-Ni NWs. Interestingly, new NiS nanoparticles are also observed on the surface of Pt-Ni NWs-S, which leads to the unique interfaces. Moreover, it is clear that the density of the newly produced nanoparticles on the surface increases with increasing the ratio of Ni to Pt in the Pt-Ni NWs. The HAADF elemental mappings of Pt-Ni NWs-S shows that, although the distribution of Pt is mainly located at the interior of NWs, Ni and S evenly distribute throughout the whole NWs (Fig. 1c,f,i,l). This result suggests that the segregated Ni in Pt-Ni NWs could be very reactive and react with the added sulfur powder to *in situ* produce NiS on the surface. In details, the high reactivity of Ni with S would drive the oxidized Ni^2+^ outward due to highly composition-segregated feature of the pristine Pt-Ni NWs. Once the interior Ni species diffuse outward, they would be captured by S species to form NiS around the NWs immediately. The successful sulfuration of Pt-Ni NWs is also confirmed by the SEM–EDS, where the S signal in Pt-Ni NWs-S is evident and the sulfuration degree increases with the increase of Ni in Pt-Ni NWs (Supplementary Fig. 5). In addition, the inductively coupled plasma–atomic emission spectroscopy (ICP–AES) result (Supplementary Table 1) reveal that the Pt/Ni ratios of Pt-Ni NWs after the sulfuration are similar to those of the pristine Pt-Ni NWs.

**Figure 2:**
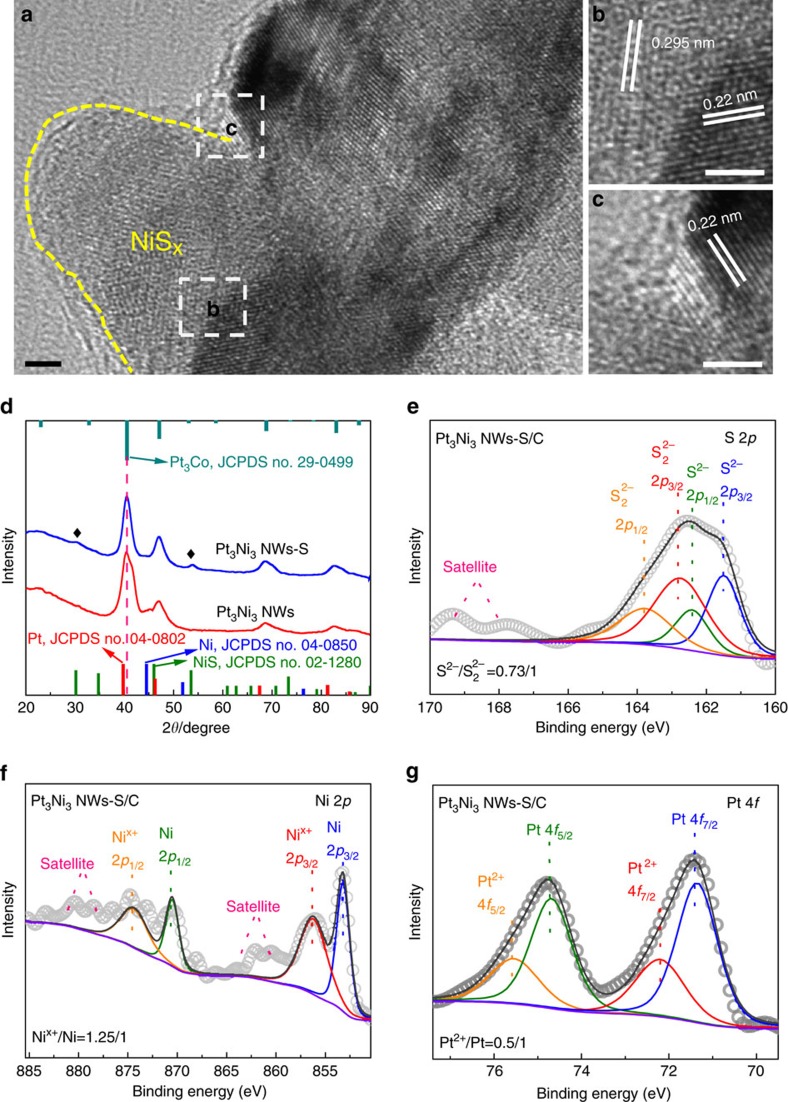
Interface and phase characterizations of Pt_3_Ni_3_ NWs-S. Representative (**a**) high-resolution TEM (HRTEM) image of Pt_3_Ni_3_ NWs-S and (**b**,**c**) the magnified HRTEM images recorded from regions b and c marked in **a**. The yellow dashed curve highlights the NiS_*x*_ particle on the surface of the Pt_3_Ni NWs. (**d**) XRD patterns of Pt_3_Ni_3_ NWs-S and Pt_3_Ni_3_ NWs. The diamond symbols denote the emerging NiS in Pt_3_Ni_3_ NWs-S. XPS patterns of (**e**) S 2*p*, (**f**) Ni 2*p* and (**g**) Pt 4*f* of Pt_3_Ni_3_ NWs-S. Scale bars, 2 nm in **a**–**c**.

The high-resolution TEM were further carried out to characterize the surface structures and interfaces between Pt-Ni NWs and NiS (Fig. 2a–c). From the Fig. 2a, the NiS nanocrystals grown on the surface of Pt_3_Ni_3_ NWs-S exhibit an intimate contact between Pt_3_Ni and NiS, forming a unique Pt_3_Ni/NiS interface. Figure 2b,c display the magnified image recorded from region b and c marked in Fig. 2a, where the lattice fringes with interplanar spacings are 0.22 and 0.295 nm, corresponding to the (111) plane of Pt_3_Ni and (100) plane of NiS, respectively. XRD analysis of Pt-Ni NWs and Pt-Ni NWs-S was used to further confirm the phase transformation after the sulfuration (Fig. 2d). It is found that the Pt_3_Ni_3_ NWs have a shoulder peak between 40.5° and 43.5° due to the highly composition-segregated feature of Pt_3_Ni_3_ NWs. After the sulfuration, the shoulder peak disappears associated with the formation of a symmetrical peak, which matches well with the *fcc* Pt_3_Ni phase in previous reports^31^,^32^,^33^ and alloyed *fcc* Pt_3_Co (Fig. 2d, JCPDS No. 29-0499). Moreover, the weak diffraction peaks of NiS are also observed in the XRD pattern of Pt_3_Ni_3_ NWs-S, which can be ascribed to the low crystalline feature of the NiS domains (Supplementary Fig. 6). Therefore, all the above results clearly indicate the transformation of the pristine Pt-Ni NWs into Pt_3_Ni/NiS heterostructures by the sulfuration process. X-ray photoelectron spectroscopy (XPS) was also used to trace the chemical states of different elements after the sulfuration (Fig. 2e–g). To be specific, the broad S 2*p* XPS peak is fitted into four peaks (Fig. 2e). The peaks located at 161.5 and 162.4 eV are attributed to the S 2*p* 3/2 and 2*p* 1/2 orbitals of divalent sulfide (S^2−^), respectively. The peak centred at 162.7 and 163.8 eV suggests the existence of bridging S_2_^2−^ (ref. 34). The ratio of S^2−^/ S_2_^2−^, calculated into 0.73/1, means that the S^2−^and S_2_^2−^ are co-existed in nickel sulfide. For the simplification, we use NiS to denote the nickel sulfide herein. Figure 2f shows Ni has both metallic and oxidized state, attributed to the NiS and Pt_3_Ni phases, respectively. XPS result reveals the Pt^0^ and Pt^2+^ are also coexisted in the Pt_3_Ni_3_ NWs-S (Fig. 2g). All the above results support that Pt_3_Ni_3_ NWs-S has been well evolved into Pt_3_Ni/NiS heterostructure after the sulfuration.

Pt-Ni NWs-S with tunable interface contact can be made by using Pt-Ni NWs with different compositions as the templates (Supplementary Figs 7–9). Except for Pt_3_Ni_1_ NWs-S, the obvious interface between NiS and Pt_3_Ni is observed in the Pt_3_Ni_2_ NWs-S and Pt_3_Ni_4_ NWs-S (Supplementary Figs 8a and 9a). Furthermore, the XRD patterns of various Pt-Ni NWs-S display similar changes to that of the Pt_3_Ni_3_ NWs-S, where the shoulder peak disappears associated with the presence of Pt_3_Ni and NiS peaks. It is worth mentioning that there are no obvious interfacial changes for Pt_3_Ni_1_ NWs after sulfuration due to the high stability of Pt_3_Ni_1_ alloy phase, as revealed by the TEM, STEM and high-resolution TEM (Supplementary Fig. 7). The presence of sulfur signals of Pt_3_Ni_1_ NWs-S in the SEM–EDS and XPS pattern are likely to be caused by the contamination of sulfur species during the sulfuration (Supplementary Fig. 7), as the sulfur signals can be largely reduced after the wash treatment of the Pt_3_Ni_1_ NWs-S (Supplementary Fig. 10). Moreover, the intensities of S^2−^ 2*p* 3/2 and 2*p* 1/2 in XPS patterns of Pt-Ni NWs-S with different compositions are gradually increased with increasing the amount of Ni, showing that high amount of Ni in Pt-Ni NWs can facilitate the formation of NiS after the sulfuration.

### HER performance of PtNi/NiS NWs

**Figure 3:**
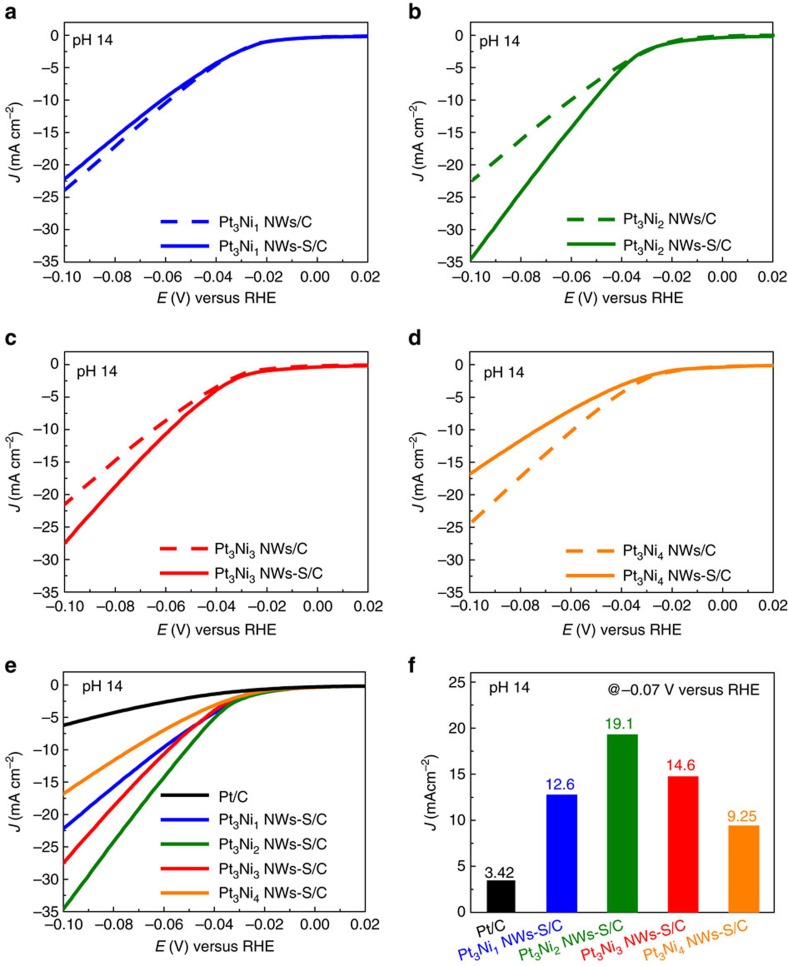
Electrocatalytic activities of different Pt-Ni NWs-S/C in the pH of 14. HER polarization curves of (**a**) Pt_3_Ni_1_ NWs-S/C and Pt_3_Ni_1_ NWs/C, (**b**) Pt_3_Ni_2_ NWs-S/C and Pt_3_Ni_2_ NWs/C, (**c**) Pt_3_Ni_3_ NWs-S/C and Pt_3_Ni_3_ NWs/C, and (**d**) Pt_3_Ni_4_ NWs-S/C and Pt_3_Ni_4_ NWs/C at pH 14, at room temperature. (**e**) HER polarization curves of different Pt-Ni NWs-S/C and Pt/C at pH of 14 at room temperature. (**f**) Histograms of current densities at −0.07 V versus RHE from **e**. All the polarization curves were recorded at a scan rate of 10 mV s^−1^ and a rotation rate of 1,600 r.p.m. with no insulation resistance compensation and all the current densities were normalized to the geometric area of working electrode.

The HER activities of pristine Pt-Ni NWs and Pt-Ni NWs-S were investigated using the linear sweep voltametry at a scan rate of 10 mV s^−1^ and room temperature without insulation resistance compensation in 1 M KOH solution. Before the HER measurements, the Pt-Ni NWs and Pt-Ni NWs-S with different compositions were uniformly loaded onto a commercial carbon black with the aid of sonication (Supplementary Figs 11 and 12). The carbon supported Pt-Ni NWs or Pt-Ni NWs-S (denoted as Pt-Ni NWs-S/C and Pt-Ni NWs/C), dispersed in a mixture of ethanol/water/Nafion solution, were dropped onto a rotating disk electrode for HER test. The loading amounts of Pt for all the catalysts were fixed at 3 μg based on the ICP–AES. Figure 3a–d shows the HER activities of Pt-Ni NWs-S/C are highly dependent on their compositions, where the sulfuration in Pt_3_Ni_1_ and Pt_3_Ni_4_ NWs does not enhance its catalytic performance for HER (Fig. 3a,d). However, the overpotentials of Pt_3_Ni_2_ NWs-S/C and Pt_3_Ni_3_ NWs-S/C for HER decrease from 60 to 51 mV and 65 to 59 mV, respectively (Fig. 3b,c). Among Pt-Ni NWs with various compositions, the Pt_3_Ni_2_ NWs-S/C shows the maximum HER activity enhancement (Supplementary Fig. 13). Furthermore, the HER activities of different Pt-Ni NWs-S/C were compared with commercial Pt/C, revealing the HER activities follow the sequence Pt_3_Ni_2_ NWs-S/C>Pt_3_Ni_3_ NWs-S/C>Pt_3_Ni_1_ NWs-S/C>Pt_3_Ni_4_ NWs-S/C>Pt/C (Fig. 3e). The current densities of these five catalysts at −0.07 V versus RHE are summarized in Fig. 3f. The current density of Pt_3_Ni_2_ NWs-S/C at −0.07 V is 19.1 mA cm^−2^, 5.6 times higher than that of the Pt/C (3.42 mA cm^−2^), demonstrating that the Pt_3_Ni_2_ NWs-S/C with unique Pt_3_Ni/NiS interface can largely promote the HER activity in alkaline medium.

**Figure 4:**
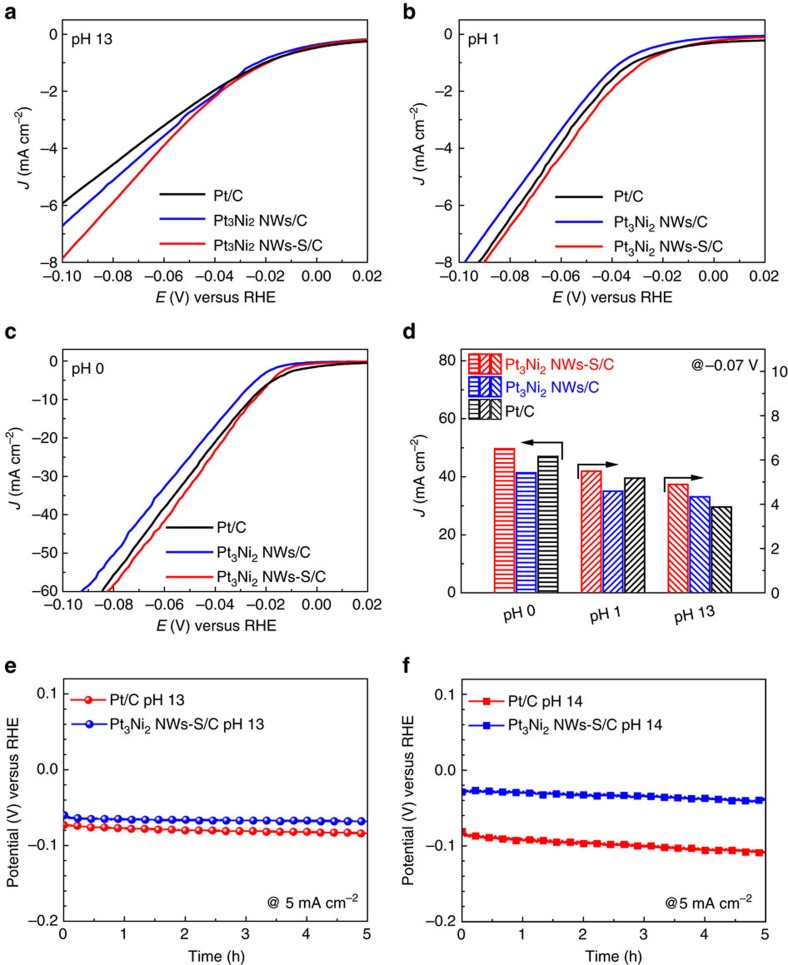
Electrocatalytic activities and stabilities of Pt_3_Ni_2_ NWs-S/C in different pH conditions. HER polarization curves of Pt_3_Ni_2_ NWs/C, Pt_3_Ni_2_ NWs-S/C and Pt/C at pH (**a**) 13, (**b**) 1 and (**c**) 0, at room temperature. (**d**) Histograms of comparative current densities at −0.07 V versus RHE from **a**–**c**. The polarization curves in **a**–**c** were recorded at a scan rate of 10 mV s^−1^ and a rotation rate of 1,600 r.p.m. with no insulation resistance compensation and the current densities were normalized to the geometric area of working electrode. Chronopotentiometry of Pt_3_Ni_2_ NWs-S/C and Pt/C under the current density of 5 mA cm−^2^ at pH (**e**) 13 and (**f**) 14 at room temperature.

Owing to the unexpected activity enhancement of Pt_3_Ni_2_ NWs-S/C for HER at pH 14, it stimulated us to explore whether it is also effective in other pH conditions. To this end, we carried out the detailed HER measurements in pH of 13 (0.1 M KOH), pH of 1 (0.05 M H_2_SO_4_) and pH of 0 (0.5 M H_2_SO_4_), respectively. The results show that the Pt_3_Ni_2_ NWs-S/C still exhibits the best HER activity among three catalysts. However, the overall activity enhancement at pH 13 is much lower than that at pH 14, suggesting that the Pt_3_Ni_2_ NWs-S/C is more susceptible at higher alkaline condition. The HER activities of Pt-Ni NWs-S/C in acidic media are also displayed in Fig. 4b,c, respectively. Similarly, the Pt_3_Ni_2_ NWs-S/C also display the best activity, but it is only slightly better than commercial Pt/C. To make a quantitative activity comparison, the current densities of Pt_3_Ni_2_ NWs-S/C, Pt_3_Ni_2_ NWs/C and Pt/C at −0.07 V at different pH are shown in Fig. 4d. It shows that the Pt_3_Ni_2_ NWs-S/C reaches the current density of 49.7, 5.50 and 4.90 mA cm^−2^ at −0.07 V at the pH 0, 1 and 13, respectively. Such activity variations of Pt-Ni NWs-S/C at different pH values demonstrate that the HER kinetics on Pt-Ni NWs-S/C is highly sensitive to the pH condition.

Other Pt-Ni NWs-S/C and Pt-Ni NWs/C with different compositions were also investigated, as shown in Supplementary Figs 14–16. The activities of both Pt_3_Ni_1_ NWs-S/C and Pt_3_Ni_4_ NWs-S/C at pH of 13 are inferior to their corresponding Pt-Ni NWs/C, similar to the trend at pH 14. In acidic condition, all the Pt-Ni NWs-S/C exhibit better activities than the Pt-Ni NWs/C and Pt/C. Comparing with the activities of Pt-Ni NWs-S/C with different compositions in different pH values (Fig. 3e and Supplementary Fig. 17), we can find that the HER activity difference of Pt-Ni NWs-S/C is more significant in alkaline condition and the activity of Pt-Ni NWs-S is more susceptible at higher alkaline condition. For this point, we suppose that it might relate to the concentration of K^+^ and the unique heterostructure between Pt_3_Ni NWs and NiS. As demonstrated by Subbaraman *et al*.^13^, the presence of hydrated cations in the vicinity of the Pt-Ni(OH)_2_ interfaces can interact with water molecules and alter the orientation of water through non-covalent interaction of Ni(OH)_2_–Li^+^–OH-H, which enhance the generation of hydrogen intermediates and thus result in the improvement of HER activity. As similar interaction of NiS–K^+^–OH-H could happen in the vicinity of Pt-Ni NWs-S, the higher HER activity of Pt-Ni NWs-S in pH of 14 than in pH of 13 can be partially ascribed to the increased concentration of K^+^. Such hypothesis has been confirmed experimentally, where in the pH of 13, the HER activity of Pt_3_Ni_3_ NWs-S increases with the addition of K^+^ (Supplementary Fig. 18). The HER activities with insulation resistance compensation at pH 13 and pH 14 were further obtained and compared with various reported results (Supplementary Fig. 19 and Supplementary Table 2). It is found that Pt_3_Ni_2_ NWs-S/C exhibits the highest activity of 37.2 and 20.2 mA cm^−2^ at pH 14 and 13 at −0.07 V among all different catalysts, respectively, 9.7 times and 2.4 times higher than those of the commercial Pt/C (3.82 mA cm^−2^ at pH 14 and 8.41 mA cm^−2^ at pH 13), also representing the highest alkaline HER activity among all the previously reported catalysts.

The HER stability of Pt_3_Ni_2_ NWs-S/C was tested using chronopotentiometry technique and further compared with the commercial Pt/C. Figure 4e shows the chronopotentiometric curves of Pt_3_Ni_2_ NWs-S/C and Pt/C at a current density of 5 mA cm^−2^ at pH 13. It shows that Pt_3_Ni_2_ NWs-S/C display the long-term stability without obvious potential shift. The stability of Pt_3_Ni_2_ NWs-S/C at pH of 14 were also tested (Fig. 4f). We can see that the HER activity loss of Pt_3_Ni_2_ NWs-S/C is less than that of Pt/C. The TEM images of Pt_3_Ni_2_ NWs-S/C after 5 h chronopotentiometry test shows that the interfaces of Pt_3_Ni_2_ NWs-S/C can be largely maintained (Supplementary Fig. 20), whereas the Pt/C has obvious agglomeration (Supplementary Fig. 21). Moreover, the SEM–EDS and XPS (Supplementary Fig. 20c,d) results reveal that the atomic ratio of Ni to S in Pt_3_Ni_2_ NWs-S/C shows almost no change after stability test. Even after the chronopotentiometry tests at very high current density and longer time, the content of S and Ni could be largely maintained (Supplementary Fig. 22), further confirming the good stability of the Pt_3_Ni_2_ NWs-S likely to be due to its larger nanostructure than that of Pt nanoparticles.

## Discussion

**Figure 5:**
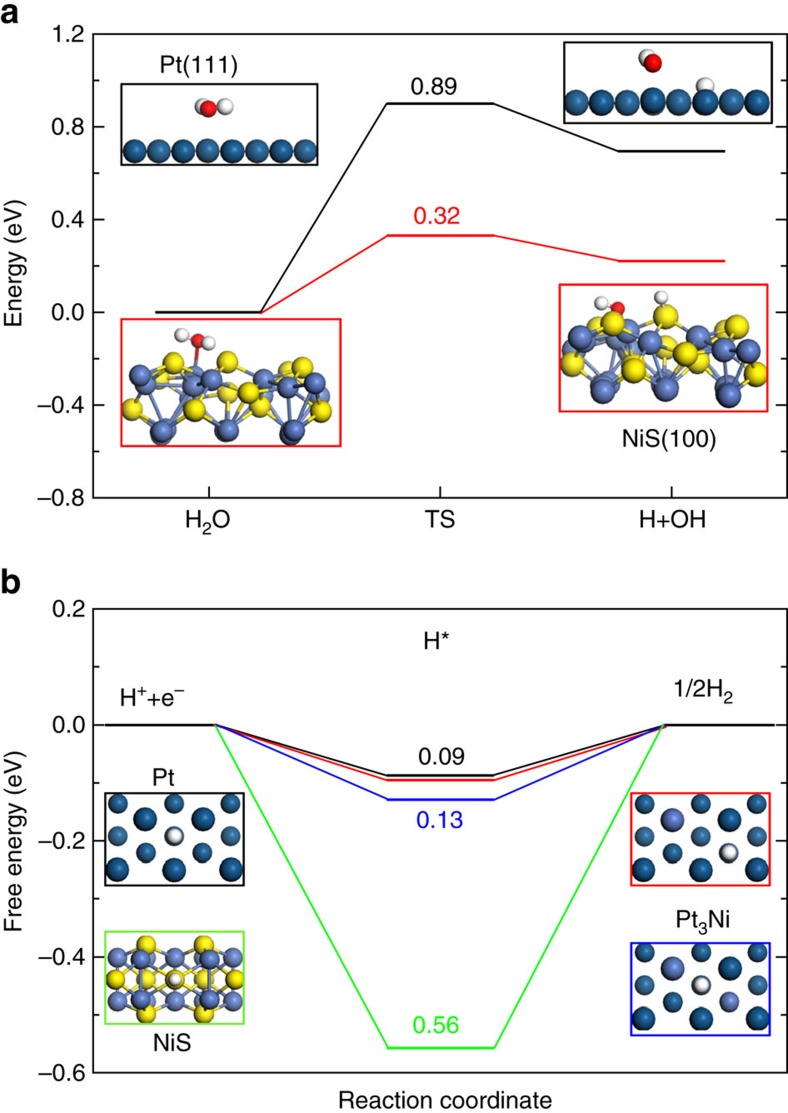
DFT simulations of HER. (**a**) Reaction energy diagram of water dissociation on the Pt (111) surface (black) and NiS (100) surface (red). The initial state (H_2_O), the transition state (TS) and the final state (H+OH) are indicated in the diagram with the corresponding energy barrier on the two surfaces. (**b**) Free-energy barriers for HER on the Pt (111) surface (black), Pt_3_Ni (111) surface (red and blue) and NiS (100) surface (green) at *U*=0 V. The red, white, yellow, dark blue and light blue spheres represent O, H, S, Pt and Ni atoms, respectively.

Here we propose that the synergy between the Pt_3_Ni and NiS components is the main factor in enhancing the alkaline HER activity. To be specific, the H_2_O in basic media would adsorb electron to be dissociated into intermediate H_ads_ and OH^−^ by NiS through the Volmer step. Owing to the unfilled *d* orbitals of Ni^2+^, the NiS can also immobilize the OH^−^ by the stronger electrostatic affinity to avoid the OH^−^ block the active site of Pt. The generated H_ads_ would be adsorbed on a nearby empty Pt site and further be converted into H_2_ readily through the Tafel step or Heyrovsky step. To examine how the individual components of NiS/Pt_3_Ni cooperate synergistically to enhance alkaline HER activity, we performed DFT calculations for the key reaction steps in alkaline HER, including the water dissociation reaction and the adsorption/combination of reactive hydrogen intermediates (H_ads_). For HER in alkaline solution, water supplies hydrogen and the dissociation of water is considered as a key rate-determining step^12^,^13^. As shown in Fig. 5a, the energy barrier for breaking the OH–H bond in water is 0.89 eV on Pt (111) surface and such a high energy barrier clearly hinders the dissociation of water to H_ads_. Strikingly, the dissociation barrier is reduced to 0.32 eV on NiS (100) surface. Thus, the NiS surface can promote water dissociation substantially and increase the rate of H_ads_ formation by orders of magnitude. Subsequently, the produced H_ads_ would combine to form H_2_ and the key quantity of interest is the free energy of hydrogen adsorption *G*_H*_ on the surface, which is often used as a descriptor for HER^35^. We calculated *G*_H*_ on Pt (111), Pt_3_Ni (111) and NiS (100) surfaces (Fig. 5b). As the hydrogen binding energy *G*_H*_ on the NiS surface is much stronger than the optimal value (*G*_H*_=0), the desorption of H_ads_ is suppressed significantly and H_2_ production is thus hindered. Consequently, the HER kinetic is sluggish on the NiS surface. In contrast, we find that the hydrogen-binding energies *G*_H*_ on Pt_3_Ni (111) surface are much smaller and closer to the optimal value; two inequivalent adsorption sites on Pt_3_Ni (111) surface (blue and red) were considered. In fact, one of the two sites (red) has almost identical *G*_H*_ as that on Pt (111), suggesting that Pt_3_Ni can be almost as efficient as Pt for the production of H_2_. It should be noted that hollowness is probably not the main reason for the HER activity enhancement, as the HER activity of the solid Pt_3_Ni_3_ NWs-S is similar to that of the Pt_3_Ni_3_ NWs-S with hollow feature (Supplementary Fig. 23). Therefore, the synergy between NiS for promoting water dissociation and Pt_3_Ni for enhancing H_ads_ adsorption and combination renders the NiS/Pt_3_Ni heterostructure as an exceptional catalyst for alkaline HER.

The synergistic effect of the Pt_3_Ni/NiS heterostructure is also responsible for the activity variations among Pt-Ni NW-S/C. For the Pt_3_Ni_1_ NWs-S/C, the synergistic effect is too weak due to the limited intimate interface between Pt_3_Ni and NiS, resulting in the stagnant dissociation of the HO-H by NiS, thus reducing the HER activity. With the proper intimate interface, Pt_3_Ni_2_ NWs-S can maximize the synergistic effect and achieve the best HER activity. However, the Pt_3_Ni_3_ NWs-S and Pt_3_Ni_4_ NWs-S with higher density of interface will make the Pt_3_Ni core be wrapped too compact to have enough active sites for Pt to absorb and recombine the H_ads_ into H_2_, leading to the gradually decreased activities. Hence, the suitable densities of NiS in Pt-Ni NWs-S/C are important factors to produce the desirable Pt_3_Ni/NiS interfaces for largely enhancing the alkaline HER activity. All the experimental and theoretical data prove that the unique interface between Pt_3_Ni NWs and NiS nanoparticles is the main factor in promoting the HER activity enhancement in the alkaline condition. However, the more detailed role of sulfide and electrocatalytic mechanism of PtNi/NiS NWs under the basic condition are still not very clear. The partial reason is that the existent corrosion and coordination effect in the basic condition might affect the apparent structure of catalysts, resulting in the electrocatalytic mechanism more complex. The *in situ* spectroscopic techniques may be good for tracking the catalytic mechanism of Pt_3_Ni/NiS interface nanowires during the HER process in the electrolytes with different pH, which will be our future endeavour.

In summary, we demonstrate a new procedure for making the Pt_3_Ni/NiS interface NWs with tunable interfaces by the direct sulfuration of highly composition-segregated Pt-Ni NWs. Owing to the optimized synergistic effects between Pt_3_Ni and NiS interface, the Pt_3_Ni_2_ NWs-S/C exhibit much enhanced performance compared with the pristine Pt_3_Ni_2_ NWs/C and the commercial Pt/C for alkaline HER. In particular, the Pt_3_Ni_2_ NWs-S/C display the best HER activity at pH 14, far exceeding the other Pt-Ni NWs-S/C. The Pt_3_Ni_2_ NWs-S/C can reach a current density of 37.2 mA cm^−2^ at 0.07 V, at pH 14, over 9.7 times higher than the commercial Pt/C (3.83 mA cm^−2^). The DFT calculations reveal that NiS could promote water dissociation to generate H^+^ while Pt_3_Ni can effectively convert H^+^ to H_2_. The synergy between the NiS and Pt_3_Ni components thus underlies the much increased HER activity in the alkaline solution. These multicomponent NWs also exhibit enhanced durability. This study highlights a novel strategy to create metal/sulfide heterostructures with excellent electrocatalytic performance for alkaline HER and beyond.

## Methods

### Chemicals

Platinum (II) acetylacetonate (Pt(acac)_2_, 97%), nickel (II) acetylacetonate (Ni(acac)_2_, 95%), hexadecyltrimethylammonium chloride (CH_3_(CH_2_)_15_N(Cl)(CH_3_)_3_, CTAC, >98.0%) and OAm (CH_3_(CH2)_7_CH=CH(CH_2_)_7_CH_2_NH_2_, 70%) were all purchased from Sigma-Aldrich. Glucose (C_6_H_12_O_6_, AR) and sulfur powder (S, CP) were purchased from Sinopharm Chemical Reagent Co. Ltd. (Shanghai, China). All the chemicals were used as received without further purification. The water (18 MΩ cm^−1^) used in all experiments was prepared by passing through an ultra-pure purification system.

### Preparation of Pt-Ni nanowires

Pt-Ni NWs were synthesized according to a previously reported method with slight modification^27^. In a typical preparation of Pt_3_Ni_*x*_ (*x*=1, 2, 3 and 4) NWs, 10 mg Pt(acac)_2_, a desirable amount of Ni(acac)_2_ (that is, 2.3, 4.6, 6.8 and 9.1), 32 mg CTAC, 60 mg glucose and 5 ml OAm were added into a vial (volume: 30 ml). After the vial had been capped, the mixture was ultrasonicated for 1 h. The resulting homogeneous mixture was then heated from room temperature to 180 °C and maintained at 180 °C for 5 h in an oil bath. After cooling to room temperature, the resulting products were collected by centrifugation and washed several times with ethanol/cyclohexane mixture.

### Preparation of Pt-Ni-S NWs

To the unpurified Pt-Ni NWs mixture at 150 °C, 3 mg sulfur powder dissolved in 1 ml OAm was added under magnetic stirring. The mixture was then kept at 150 °C for another 3 h. The final products were collected by centrifugation and washed three times with an ethanol/cyclohexane mixture.

### Characterization

TEM and HAADF–STEM images were conducted on an FEI Tecnai F20 TEM at an acceleration voltage of 200 kV. SEM images were taken with a HITACHI S-4700 cold field-emission SEM operated at 15 kV. The samples were prepared by dropping cyclohexane dispersion of samples onto carbon-coated copper TEM grids using pipettes and dried under ambient condition. PXRD patterns were collected on X'Pert-Pro MPD diffractometer (Netherlands PANalytical) with a Cu Kα X-ray source (*λ*=1.540598 Å). The concentrations of catalysts were determined by the ICP–AES (710-ES, Varian, ICP–AES). XPS was carried out on an SSI S-Probe XPS Spectrometer. The carbon peak at 284.6 eV was used as a reference to correct for charging effects.

### Electrochemical measurements

Electrochemical measurements were performed by using CHI660 electrochemical workstation (Chenhua, Shanghai). A three-electrode cell was used to perform the electrochemical measurements. The working electrode was a rotating disk electrode (RDE) (diameter: 5 mm, area: 0.196 cm^2^) from Pine Instruments. A saturated calomel electrode and platinum wire were used as the reference and counter electrodes, respectively. The catalyst ink was prepared by ultrasonically mixing catalysts with 895 μl of ethanol, 100 μl of water and 5 μl of 5 wt% Nafion solutions for 1 h. The actual mass concentrations of the Pt species in all catalyst inks were controlled at 0.30 mg_Pt_ per ml. Then, 10 and 50 μl of the ink were deposited on RDE dried at room temperature, to obtain the working electrodes for activity and stability test, respectively. The loading of Pt on the working electrodes were 15.3 and 76.5 μg cm^−2^ for activity test and stability test, respectively.

HER measurements were conducted in pH values of 14, 13, 1 and 0. All the fresh electrolytes with required concentrations were prepared in a volumetric flask by dissolving the required amount of KOH or H_2_SO_4_ in Milli-Q water and purged with N_2_ before the measurement. During the measurement, RDE electrode was constantly rotating at 1,600 r.p.m. to get rid of the bubbles. Linear sweep voltammetry was carried out between −0.2 and 0.2 V at the scan rate of 10 mV s^−1^ for activity tests. The catalysts were cycled about 20 cycles at the scan rate of 100 mV s^−1^ between −0.2 and 0.2 V until a stable cyclic voltammetry curve was developed before linear sweep voltammetry test. The chronopotentiometry was measured under a constant current density of 5 mA cm^−2^ for stability test.

For comparison, the commercial Pt/C (20% loading, 2–5 nm Pt size, Johnson Matthey) was used as the baseline catalyst with the same loading amount of Pt at 15.3 μg cm^−2^ for activity test and 76.5 μg cm^−2^ for stability test. The polarization curves with or without insulation resistance compensation are mentioned in the paper. All electrochemical measurements were performed at room temperature.

### DFT models and calculations

Spin-polarized DFT calculations were carried out using the VASP package^36^,^37^ with the projector-augmented wave pseudopotentials^38^ and Perdew–Burke–Ernzerhof generalized gradient approximation^39^. An energy cutoff of 400 eV was used for the plane-wave basis set. The Pt (111), Pt_3_Ni (111) and NiS (100) surfaces were modelled by periodically repeated four-layer slabs with (3 × 3) unit cells. The Brillouin zone was sampled with the Monkhorst–Pack scheme^40^ and 3 × 3 × 1 k-point mesh. The atoms in the top three layers were fully relaxed, while the rest of the atoms were fixed in their equilibrium positions. The force convergence criterion for atomic relaxation was 0.02 eV Å^−1^.

To determine the transition state for water dissociation on the Pt (111) and NiS (100) surfaces, we employed the climbing image nudged elastic band method^41^. Furthermore, the force convergence tolerance on each atom in search of the minimum energy path was set to be 0.05 eV Å^−1^. The free energy diagram for HER was obtained by calculating the change of the free energy with a hydrogen atom adsorbed on the surface following the computational hydrogen electrode model^35^. The hydrogen adsorption free energy *G*_H*_ is determined as *G*_H*_=*E*[surf+H]−*E*[surf]−*E*[H_2_]/2+Δ*E*_ZPE_−*T*Δ*S*, where *E*[surf +H] and *E*[surf] are the total energies of the surface with and without the H adsorbate, respectively. *E*[H_2_] is the total energy of a hydrogen molecule. Δ*E*_ZPE_ is the difference in the zero-point energy between the adsorbed H atom and the gaseous phase H_2_; Δ*S* is the difference in entropy. At *T*=300 K, *G*_H*_ can be calculated by G_H*_=*E*[surf+H]−*E*[surf]−*E*[H_2_]/2+0.24 eV^35^.

### Data availability

All relevant data are available from the authors on request.

## Additional information

**How to cite this article:** Wang, P. *et al*. Precise tuning in platinum-nickel/nickel sulfide interface nanowires for synergistic hydrogen evolution catalysis. *Nat. Commun.* **8**, 14580 doi: 10.1038/ncomms14580 (2017).

**Publisher's note**: Springer Nature remains neutral with regard to jurisdictional claims in published maps and institutional affiliations.

## Supplementary Material

Supplementary Information Supplementary Figures and Supplementary Tables
