# Supplementary material for: Precise tuning in platinum-nickel/nickel sulfide interface nanowires for synergistic hydrogen evolution catalysis
Source: Nat Commun. 2017 Feb 27;8:14580. doi: 10.1038/ncomms14580 (PMC5333357; doi:10.1038/ncomms14580)
Supplement: Supplementary Information — Supplementary Figures and Supplementary Tables [file ncomms14580-s1.pdf]

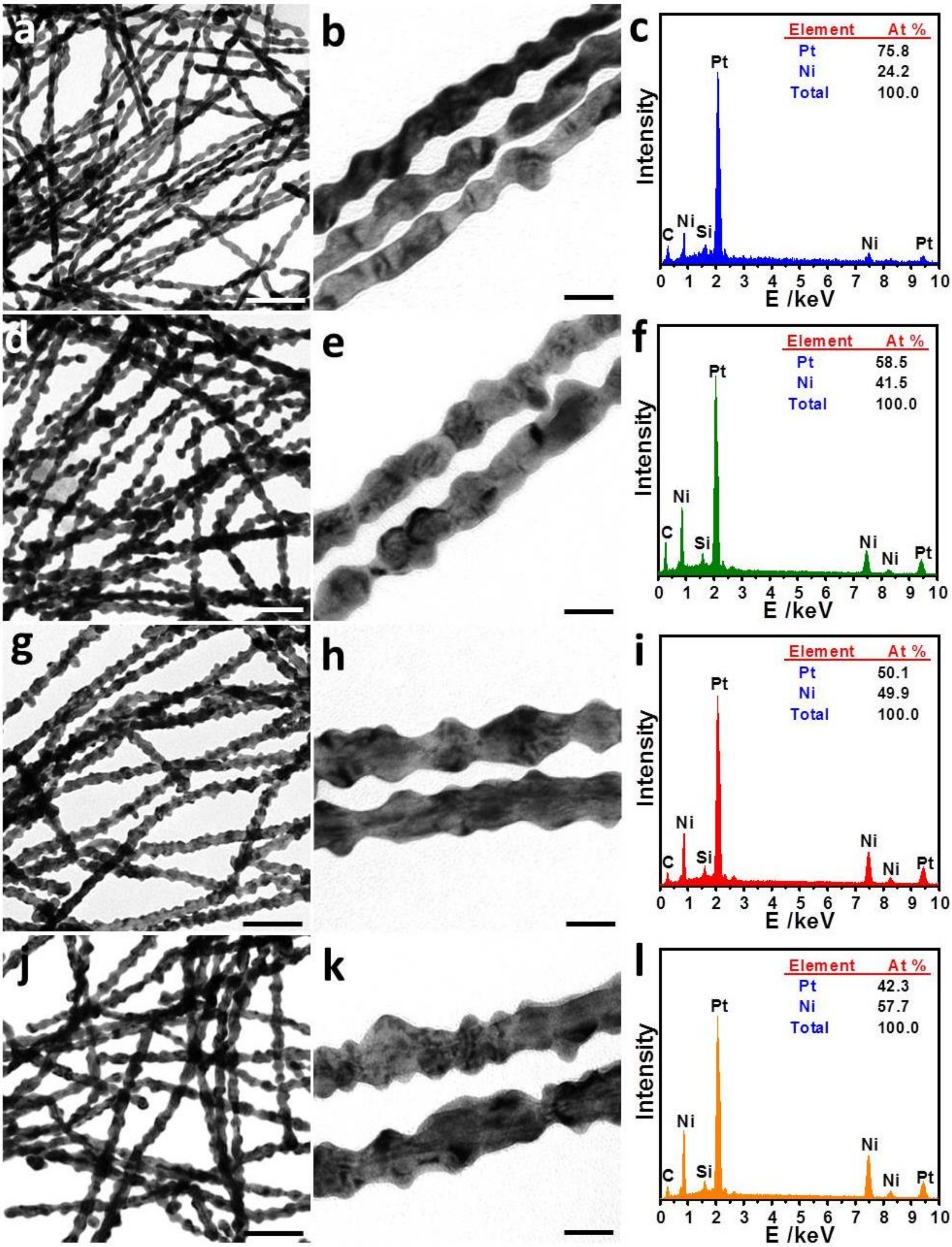

Supplementary Figure 1. TEM images of pristine (a, b) Pt_3Ni_1 NWs, (d, e) Pt_3Ni_2 NWs, (g, h) Pt_3Ni_3 NWs and (j, k) Pt_3Ni_4 NWs. SEM-EDS patterns of pristine (c) Pt_3Ni_1 NWs, (f) Pt_3Ni_2 NWs, (i) Pt_3Ni_3 NWs and (l) Pt_3Ni_4 NWs. The scale bars in a, d, g and j are 100 nm. The scale bars in b, e, h and k are 20 nm.

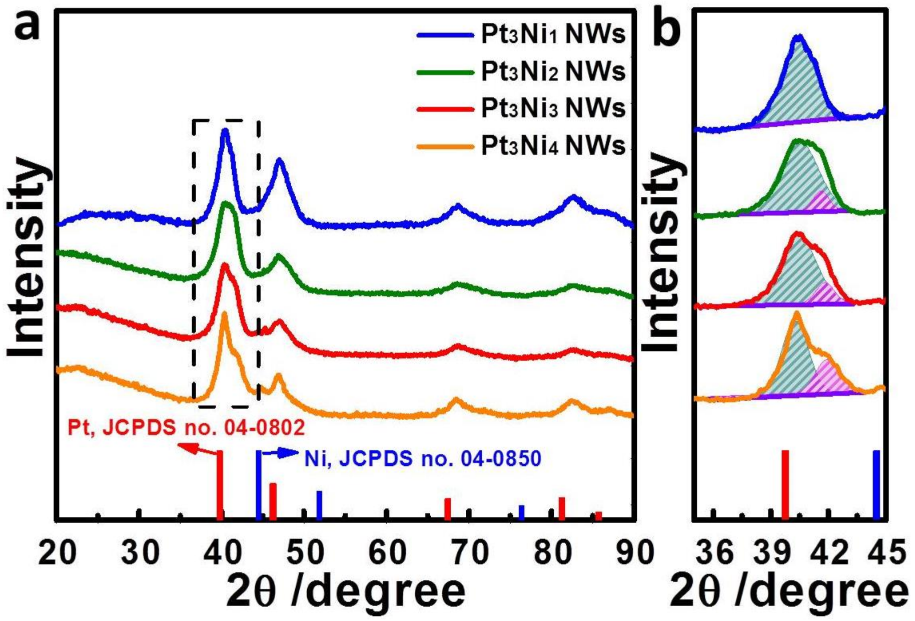

Supplementary Figure 2. PXRD patterns of pristine Pt_3Ni_1 NWs, Pt_3Ni_2 NWs, Pt_3Ni_3 NWs and Pt_3Ni_4 NWs.

(b) The fitted peak curves from the dashed region marked in (a).

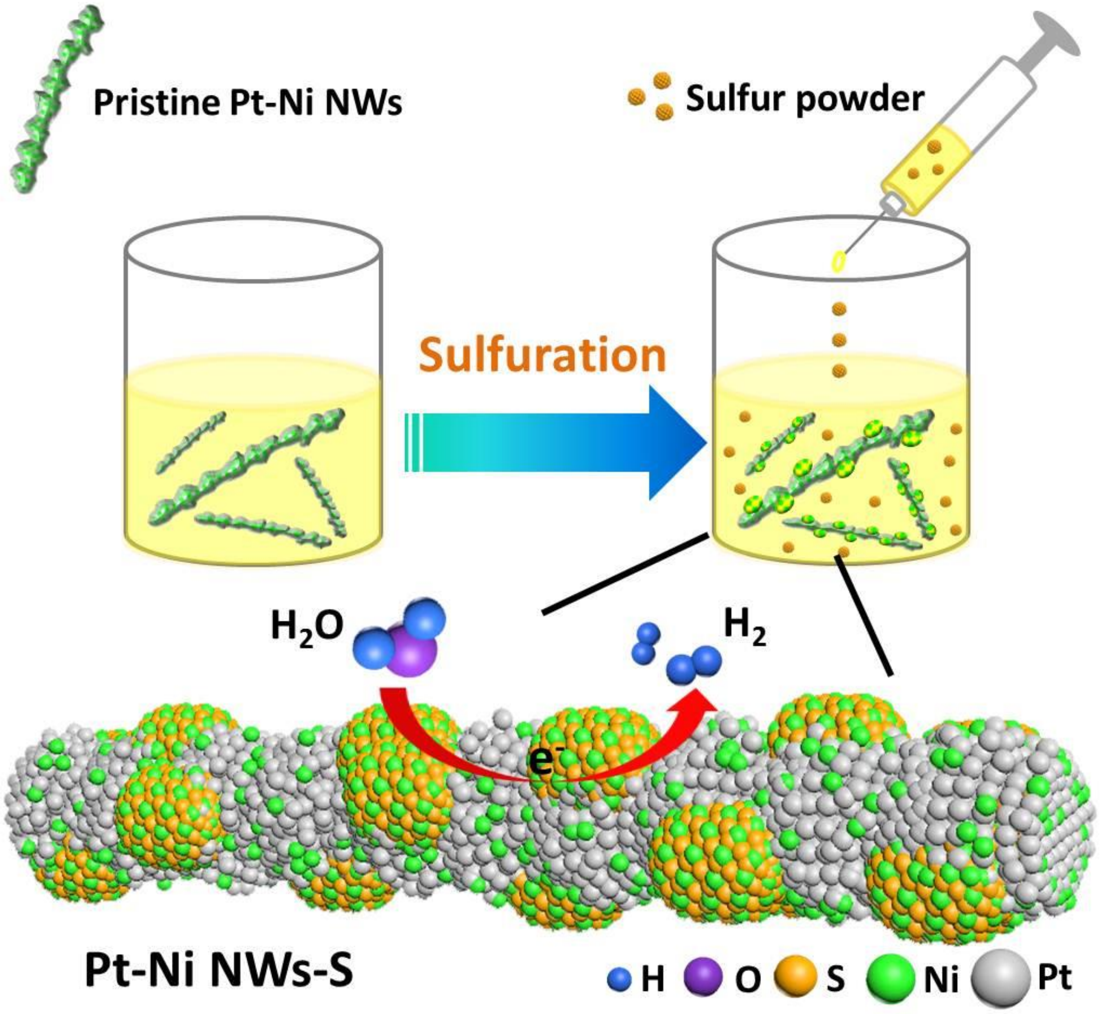

Supplementary Figure 3. Schematic illustration on the sulfuration of the composition-segregated Pt-Ni NWs. The sulfuration was performed by adding sulfur powder into the pristine Pt-Ni NWs to achieve the Pt-Ni NWs-S with heterostructures.

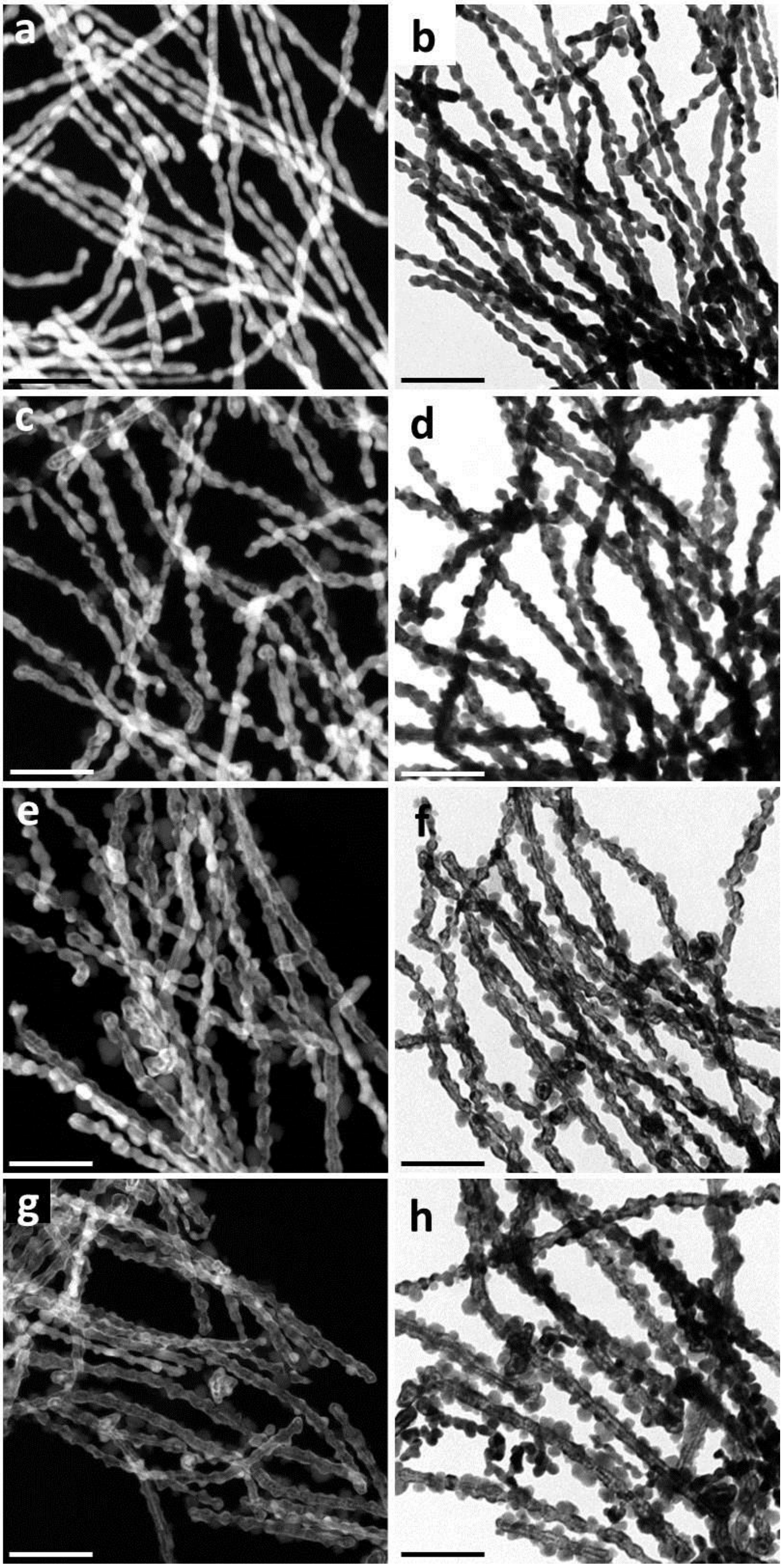

Supplementary Figure 4. Additional HAADF-STEM images and TEM images of (a, b) Pt_3Ni_1 NWs-S, (c, d) Pt_3Ni_2 NWs-S, (e, f) Pt_3Ni_3 NWs-S and (g, h) Pt_3Ni_4 NWs-S. The scale bars in a-h are 100 nm.

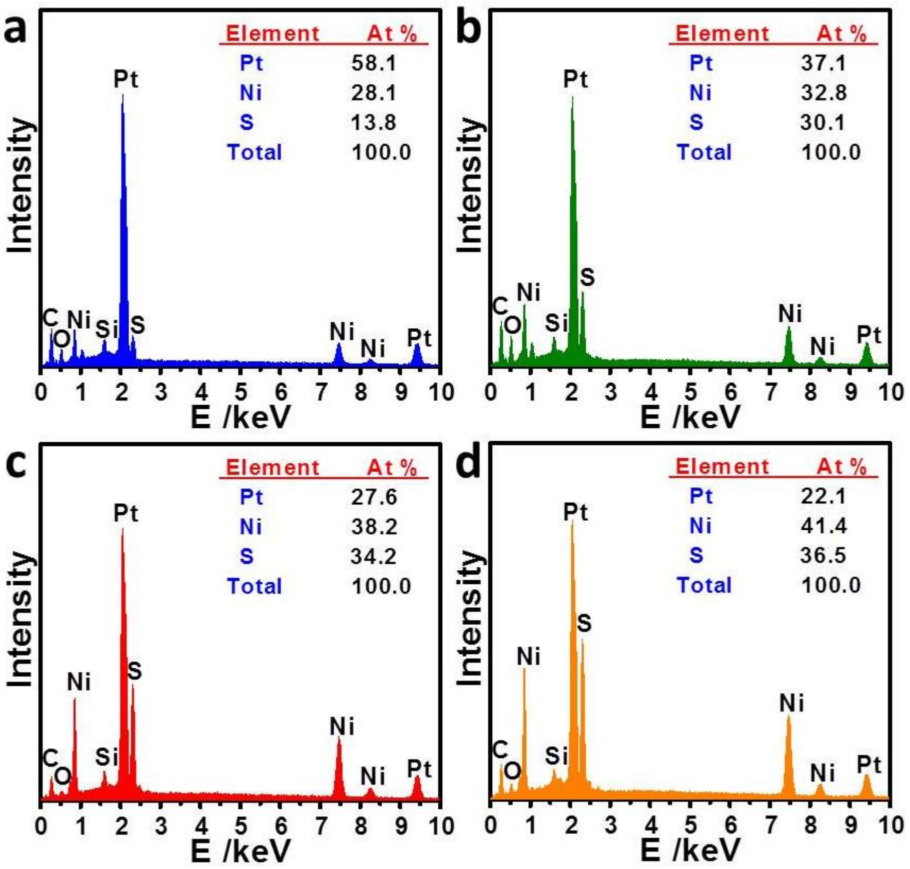

Supplementary Figure 5. SEM-EDS patterns of (a) Pt_3Ni_1 NWs-S, (b) Pt_3Ni_2 NWs-S, (c) Pt_3Ni_3 NWs-S and (d) Pt_3Ni_4 NWs-S.

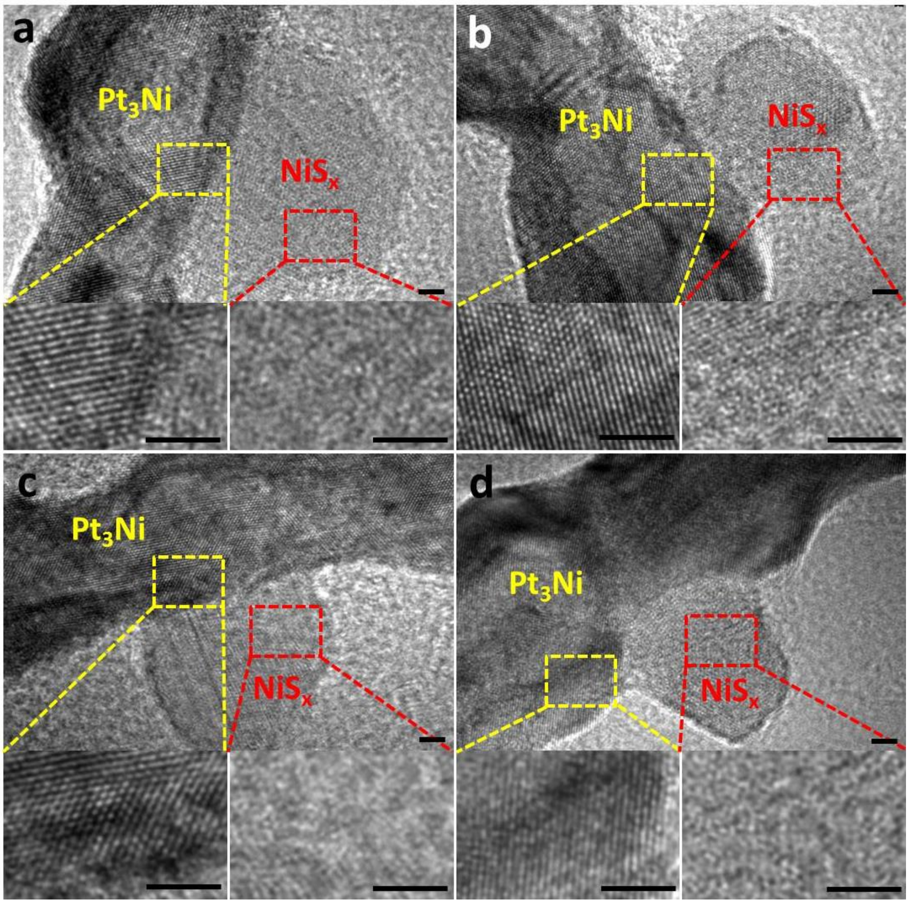

Supplementary Figure 6. Additional HRTEM images of (a, b, c, d) Pt_3Ni_3 NWs-S. The scale bars in a-d are 2 nm.

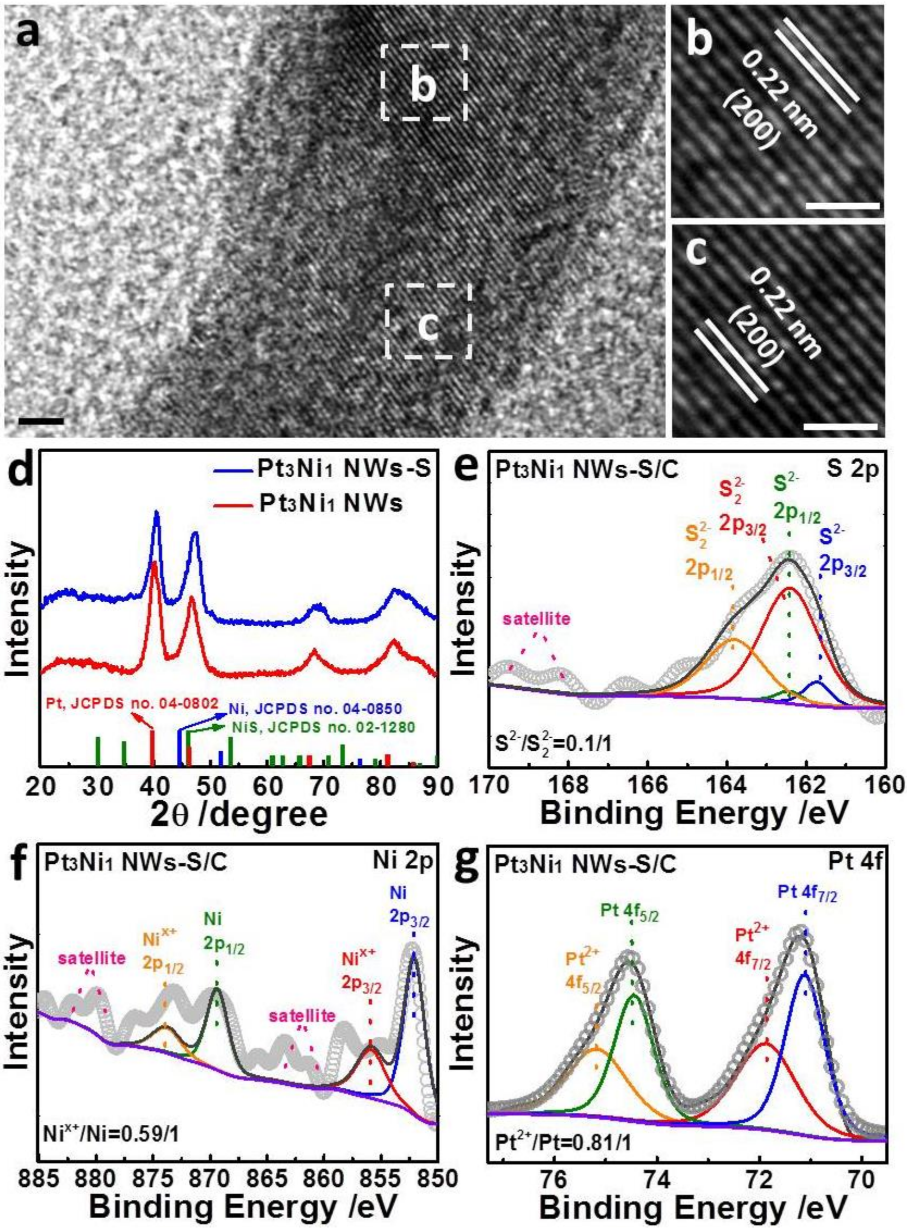

Supplementary Figure 7. (a) HRTEM image of Pt_3Ni_1 NWs-S and (b, c) the magnified HRTEM images recorded from regions b and c marked in (a). (d) XRD patterns of Pt_3Ni_1 NWs-S and Pt_3Ni_1 NWs. XPS patterns of (e) S 2p, (f) Ni 2p and (g) Pt 4f of Pt_3Ni_1 NWs-S. The scale bars in a-c are 2 nm.

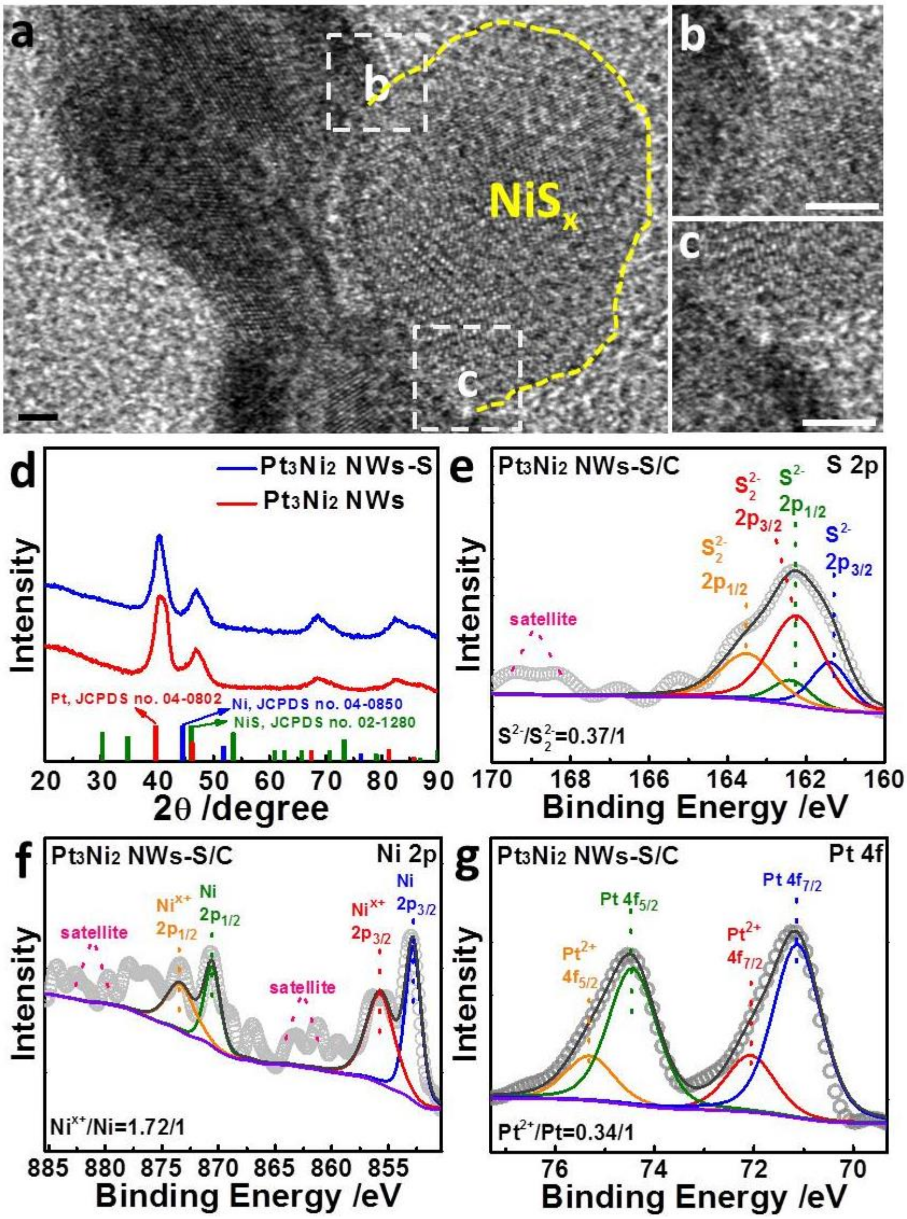

Supplementary Figure 8. (a) HRTEM image of Pt_3Ni_2 NWs-S and (b, c) the magnified HRTEM images recorded from regions b and c marked in (a). (d) XRD patterns of Pt_3Ni_2 NWs-S and Pt_3Ni_2 NWs. XPS patterns of (e) S 2p, (f) Ni 2p and (g) Pt 4f of Pt_3Ni_2 NWs-S. The scale bars in a-c are 2 nm.

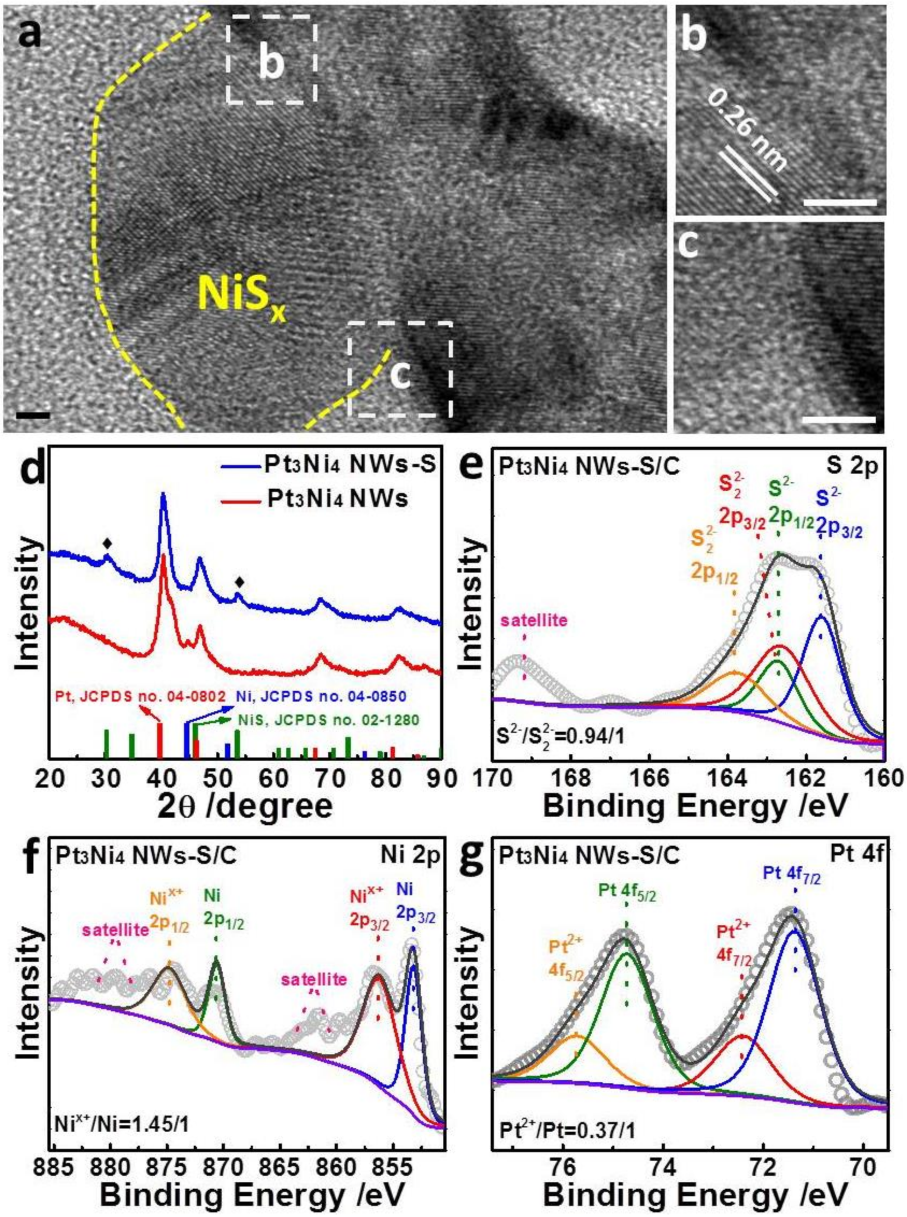

Supplementary Figure 9. (a) HRTEM image of Pt_3Ni_4 NWs-S and (b, c) the magnified HRTEM images recorded from regions b and c marked in (a). (d) XRD patterns of Pt_3Ni_4 NWs-S and Pt_3Ni_4 NWs. XPS patterns of (e) S 2p, (f) Ni 2p and (g) Pt 4f of Pt_3Ni_4 NWs-S. The scale bars in a-c are 2 nm.

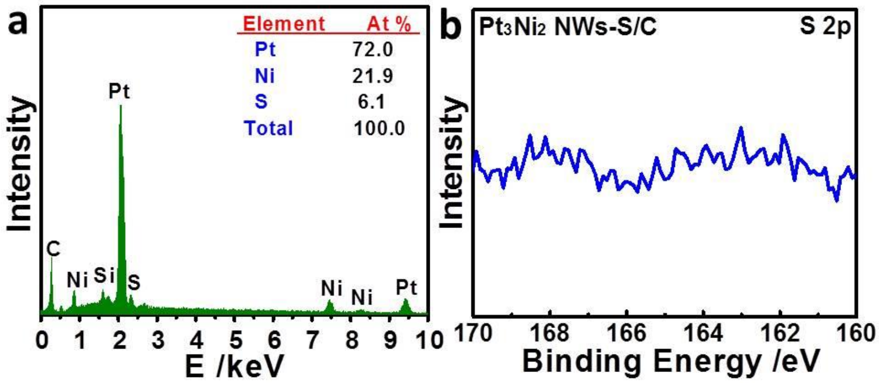

Supplementary Figure 10. (a) SEM-EDS and (b) S 2p XPS spectrum of Pt_3Ni_1 NWs-S after their washed with CS_2 and ethanol to remove the adsorbed sulfur on NW surface.

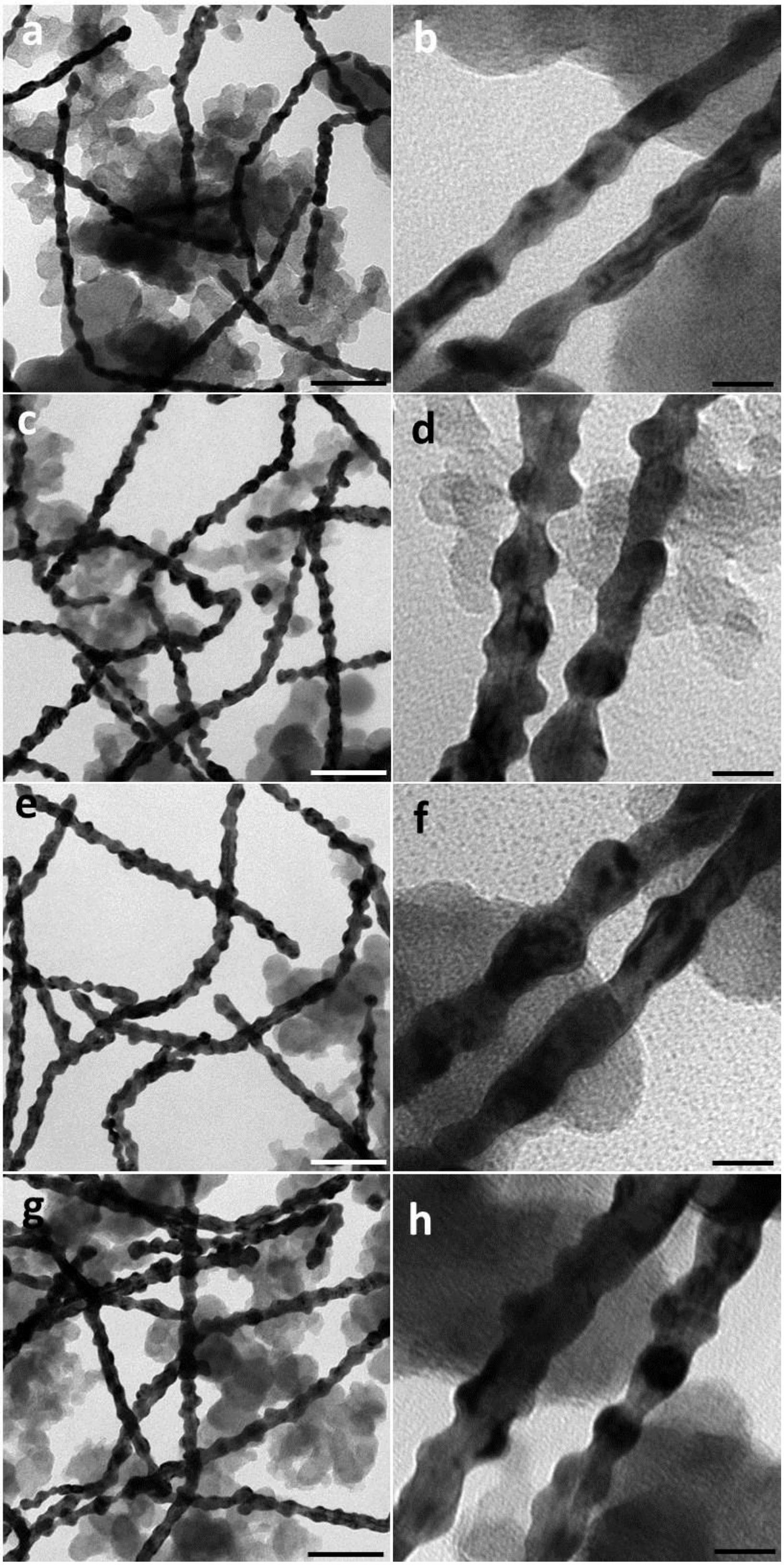

Supplementary Figure 11. TEM images of (a) Pt_3Ni_1 NWs/C, (b) Pt_3Ni_2 NWs/C, (c) Pt_3Ni_3 NWs/C and (d) Pt_3Ni_4 NWs/C. The scale bars in a, c, e and g are 100 nm. The scale bars in b, d, f and h are 20 nm.

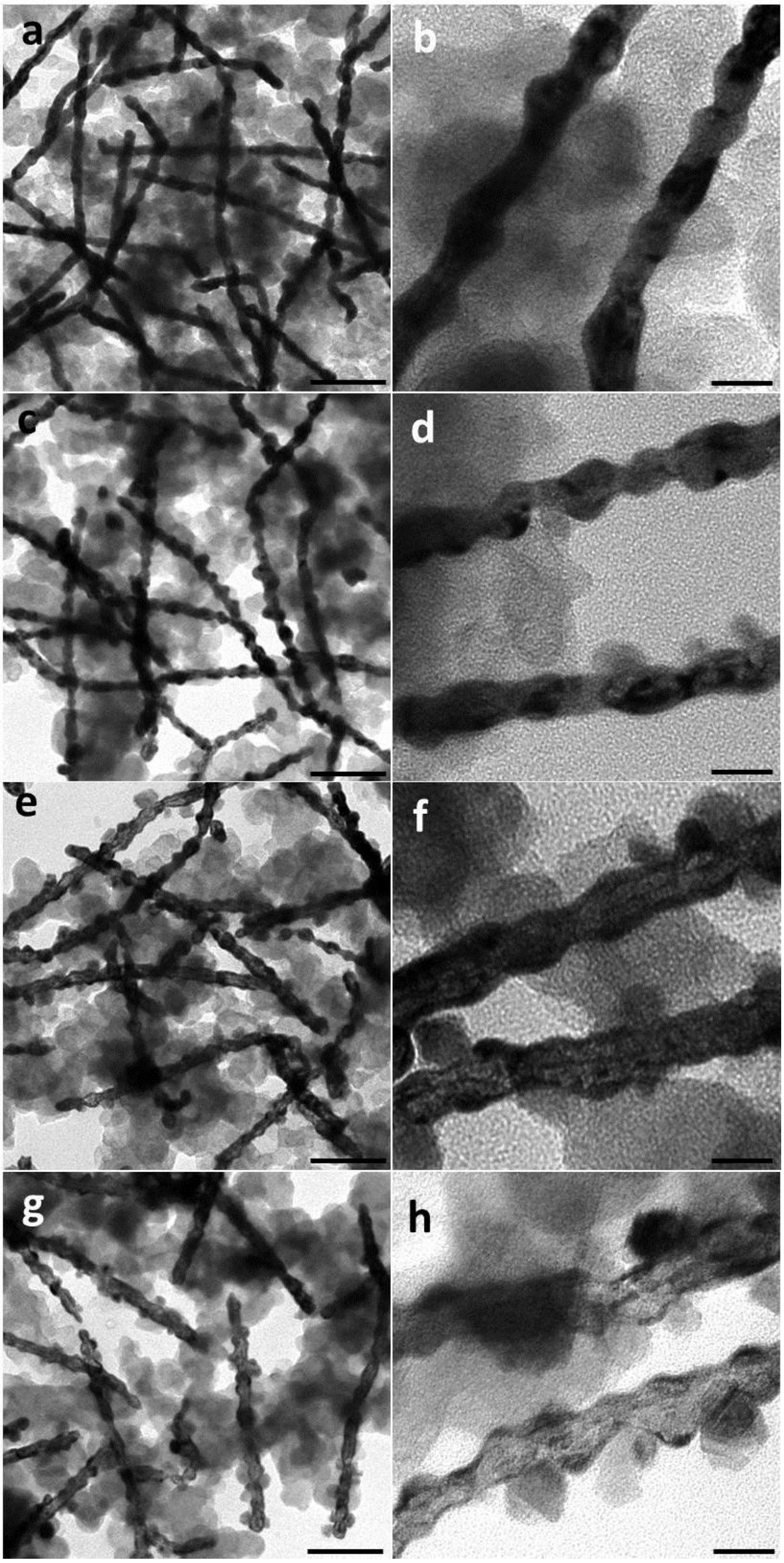

Supplementary Figure 12. TEM images of (a, b) Pt_3Ni_1 NWs-S/C , (c, d) Pt_3Ni_2 NWs-S/C, (e, f) Pt_3Ni_3 NWs-S/C and (g, h) Pt_3Ni_4 NWs-S/C. The scale bars in a, c, e and g are 100 nm. The scale bars in b, d, f and h are 20 nm.

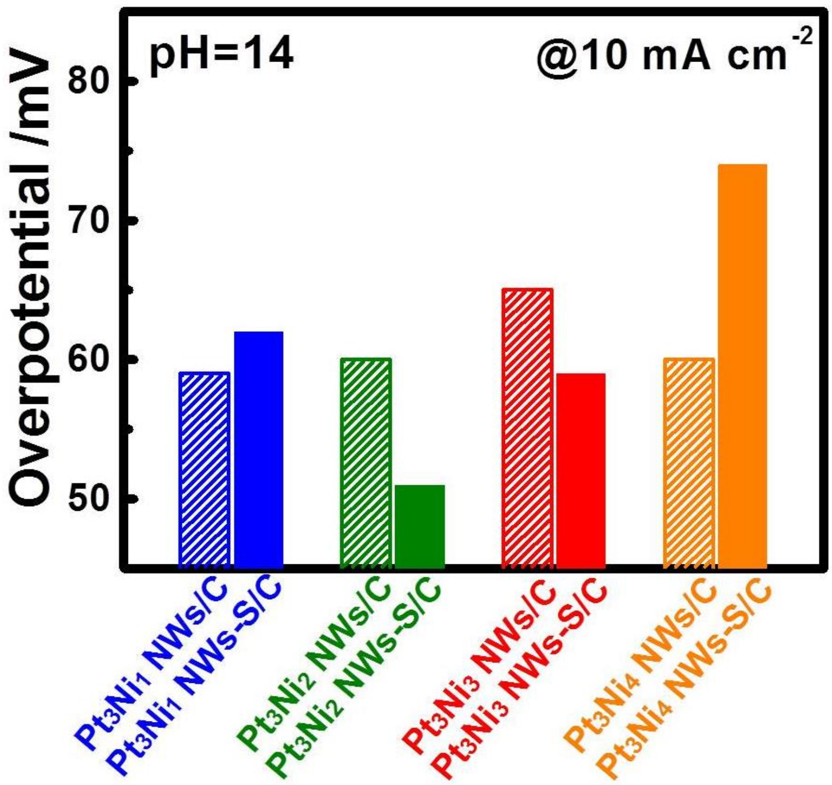

Supplementary Figure 13. The overpotential of different Pt-Ni NWs/C and corresponding Pt-Ni NWs-S/C at the current density of 10 mA cm^{-2}.

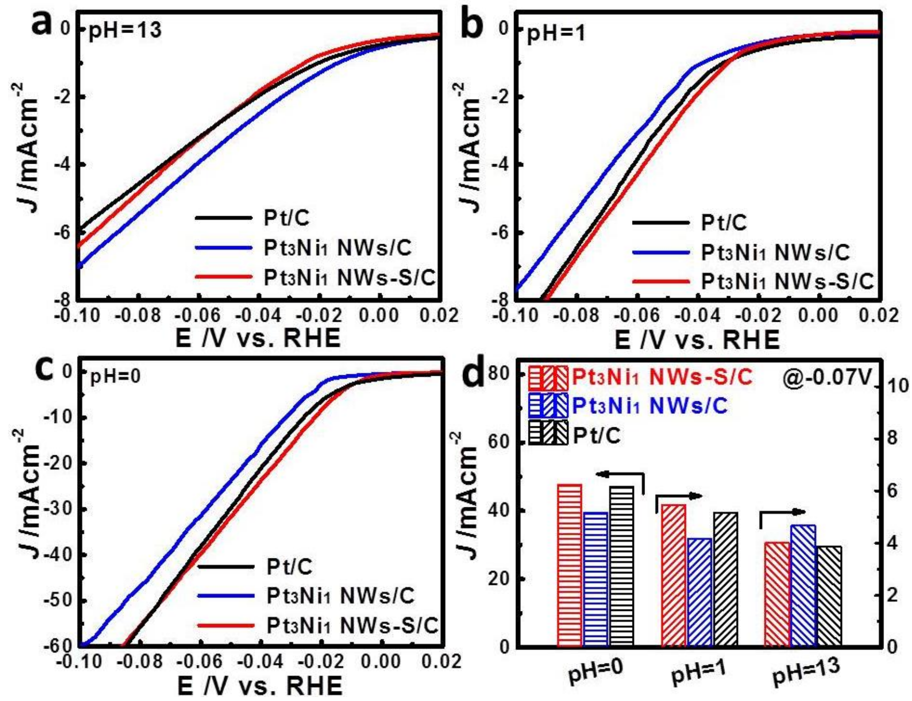

Supplementary Figure 14. HER polarization curves of Pt_3Ni_1 NWs/C, Pt_3Ni_1 NWs-S/C and Pt/C at pH of (a) 13, (b) 1 and (c) 0. (d) Histograms of current densities at -0.07 V *versus* RHE from (a)-(c).

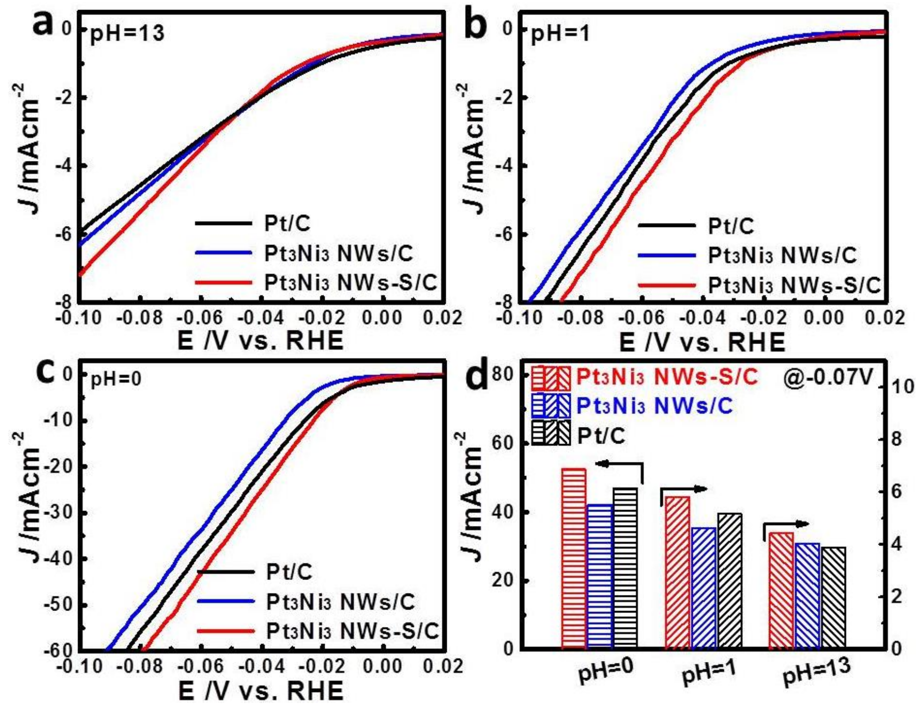

Supplementary Figure 15. HER polarization curve of Pt_3Ni_3 NWs/C, Pt_3Ni_3 NWs-S/C and Pt/C at pH of (a) 13, (b) 1 and (c) 0. (d) Histograms of current densities at -0.07 V *versus* RHE from (a)-(c).

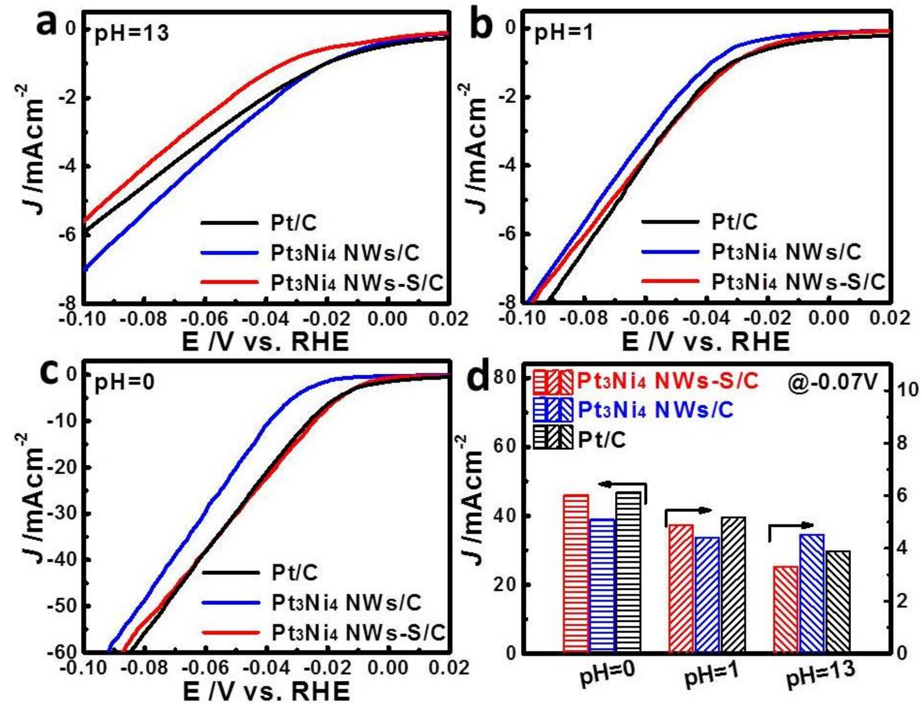

Supplementary Figure 16. HER polarization curve of Pt_3Ni_4 NWs/C, Pt_3Ni_4 NWs-S/C and Pt/C at pH of (a) 13, (b) 1 and (c) 0. (d) Histograms of current densities at -0.07 V *versus* RHE from (a)-(c).

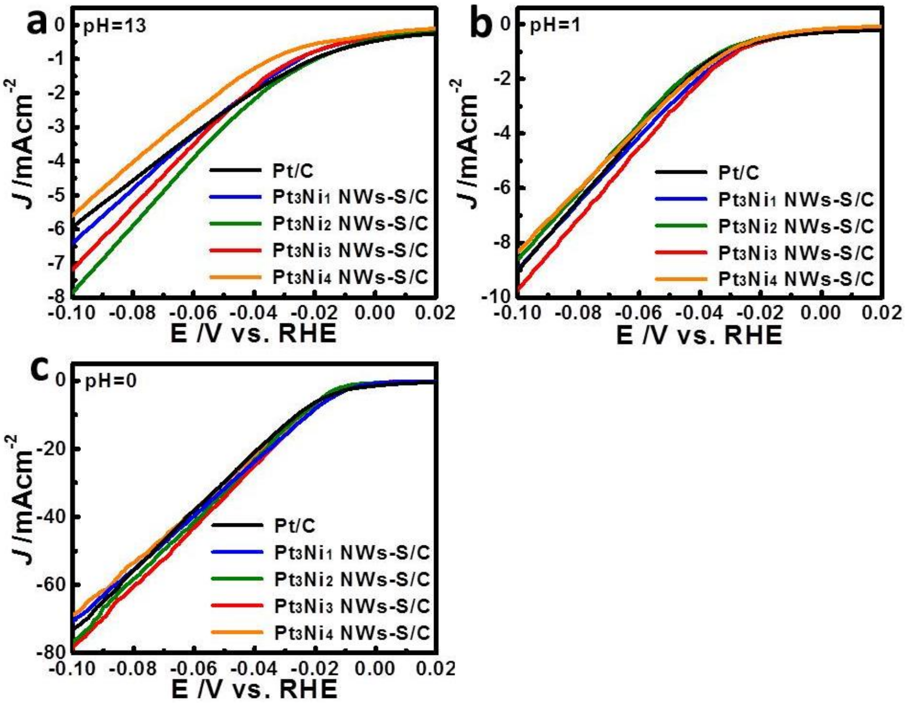

Supplementary Figure 17. HER polarization curves of Pt-Ni NWs-S /C with different compositions and Pt/C at pH of (a) 13, (b) 1 and (c) 0.

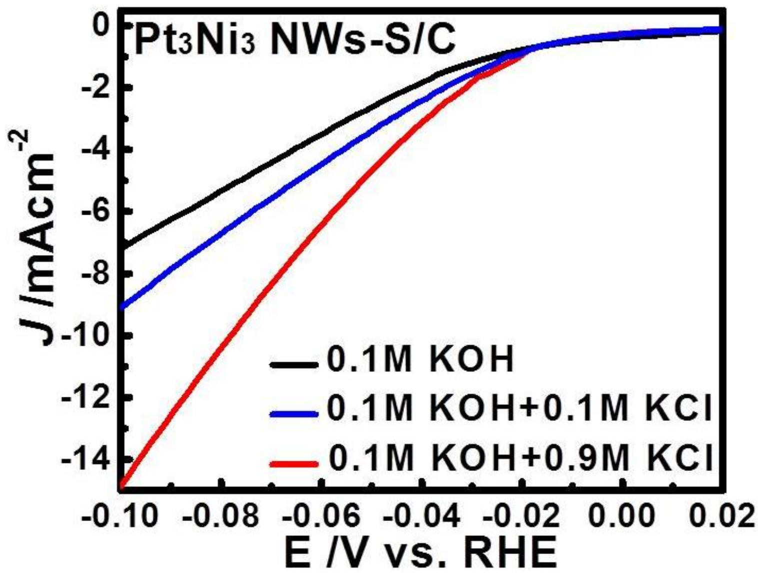

Supplementary Figure 18. HER activities of Pt_3Ni_3 NWs-S/C in pH of 13 with different concentrations of K^+.

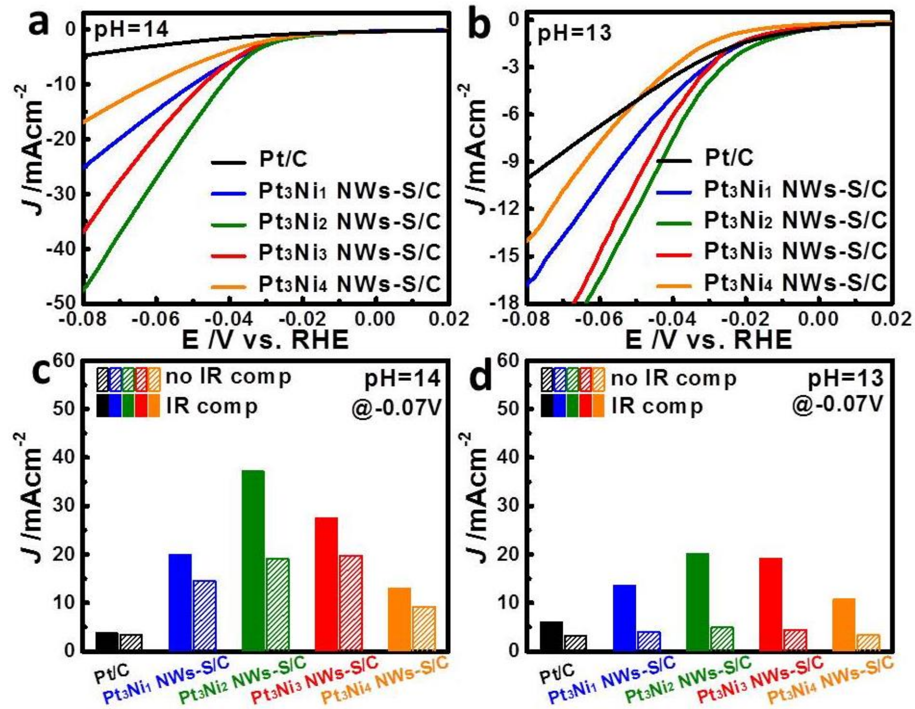

Supplementary Figure 19. HER polarization curves of Pt_3Ni_1 NWs-S/C, Pt_3Ni_2 NWs-S/C, Pt_3Ni_3 NWs-S/C, Pt_3Ni_4 NWs-S/C and Pt/C at pH of (a) 14 and (b) 13. Histograms of current densities at -0.07 V *versus* RHE with IR compensation or no IR compensation at pH of (c) 14 and (d) 13.

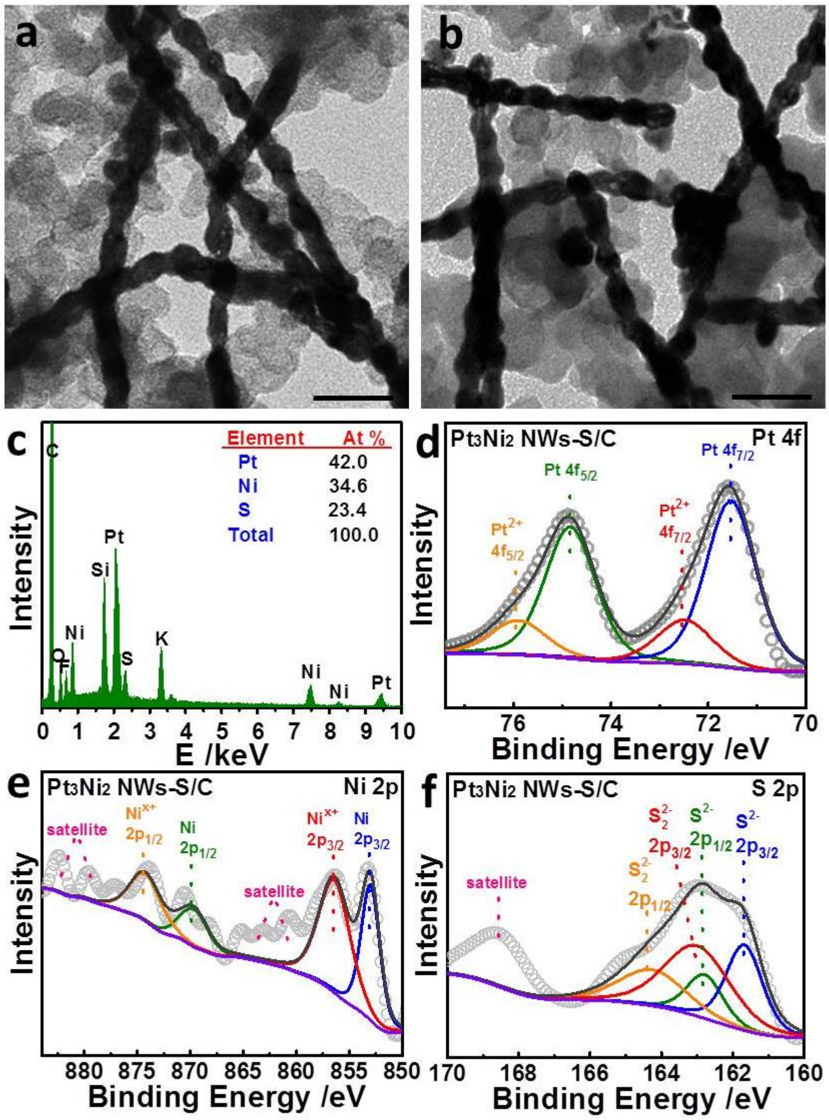

Supplementary Figure 20. TEM images of Pt_3Ni_2 NWs-S/C after 5 h chronopotentiometry test in (a) 0.1 M KOH and (b) 1 M KOH. (c) SEM-EDS and (d-f) XPS patterns of Pt_3Ni_2 NWs-S/C after 5 h chronopotentiometry test in 1 M KOH. (d) Pt 4f, (e) Ni 2p and (f) S 2p. The scale bars in a and b are 50 nm.

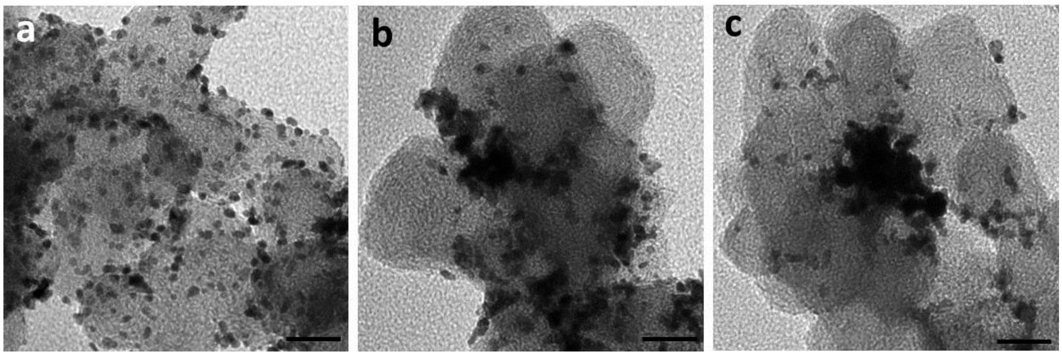

Supplementary Figure 21. TEM images of Pt/C catalyst (a) and after 5 h chronopotentiometry test in (b) 0.1M KOH and (c) 1M KOH. The scale bars in a-c are 20 nm.

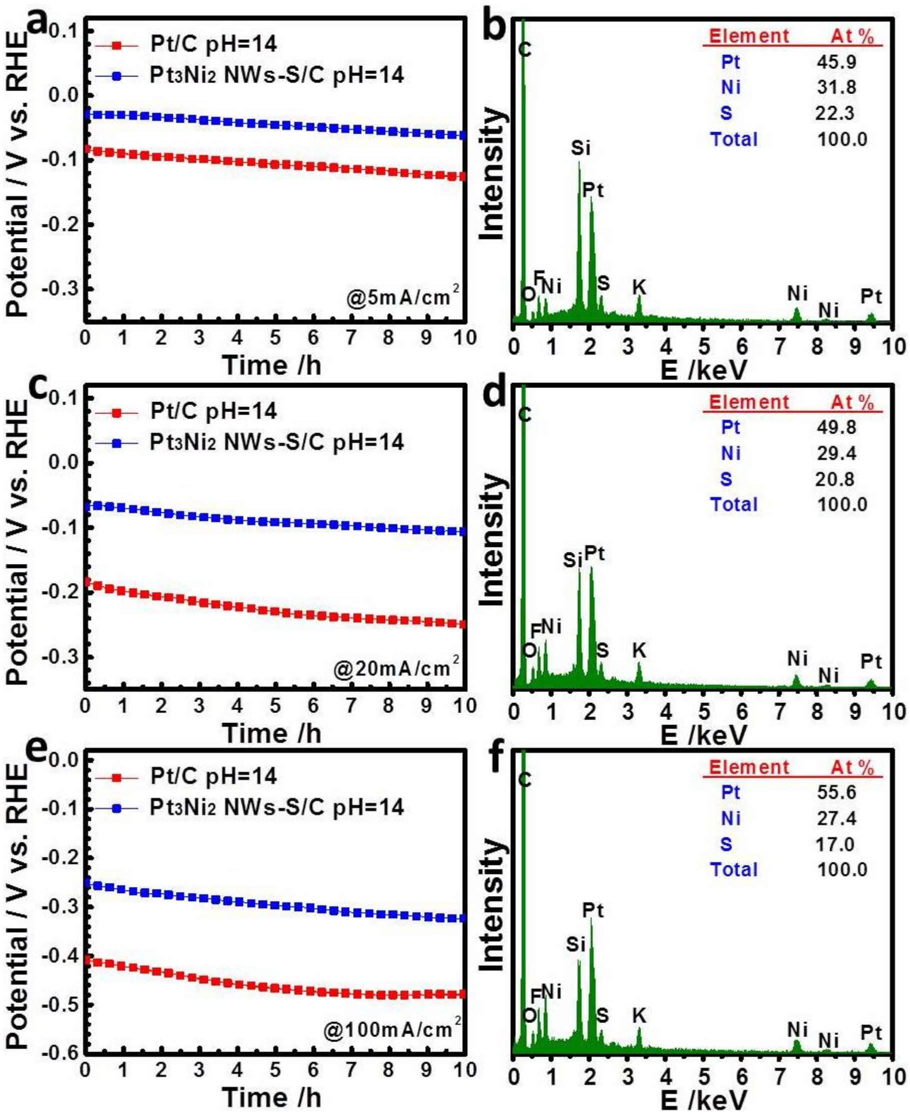

Supplementary Figure 22. Chronopotentiometry of Pt_3Ni_2 NWs-S/C and Pt/C at current densities of (a) 5 mA/cm^2, (c) 20 mA/cm^2 and (e) 100 mA/cm^2 at pH of 14. SEM-EDS patterns of Pt_3Ni_2 NWs-S/C after 10 h chronopotentiometry test at pH of 14 at current densities of (b) 5 mA/cm^2, (d) 20 mA/cm^2 and (f) 100 mA/cm^2.

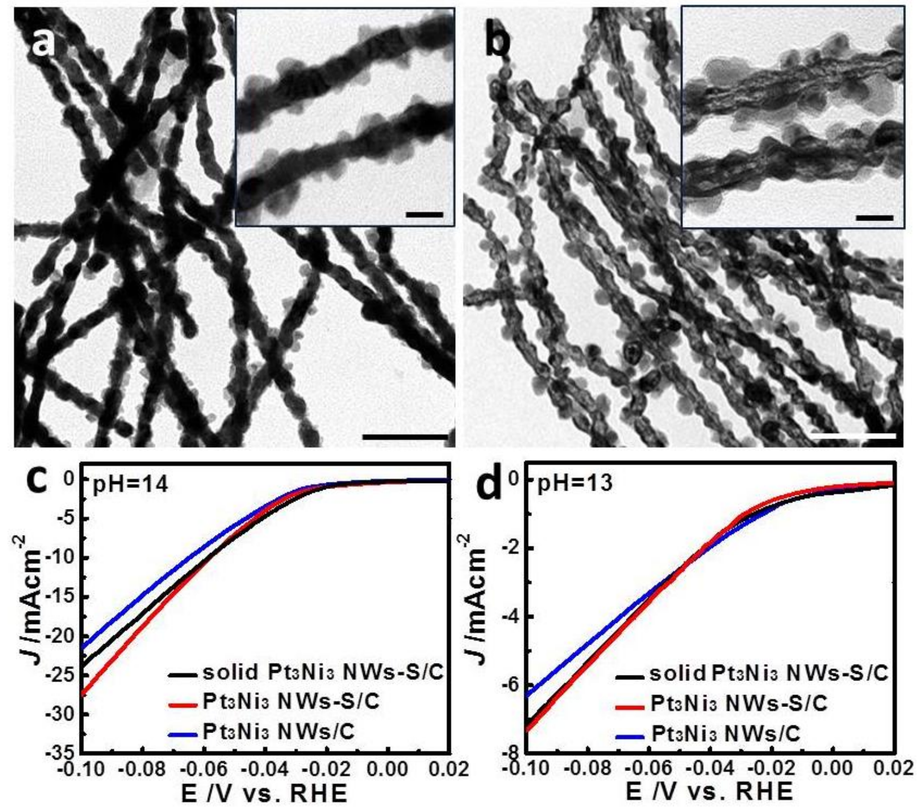

Supplementary Figure 23. TEM images of (a) solid Pt_3Ni_3 NWs-S and (b) Pt_3Ni_3 NWs-S. The inset images of (a) and (b) are corresponding high-magnification TEM images. HER activities of solid Pt_3Ni_3 NWs-S/C, Pt_3Ni_3 NWs-S/C and Pt_3Ni_3 NWs/C in pH of (c) 14 and (d) 13. The scale bars in a, b and inset of a, b are 100 nm and 20 nm, respectively.

Supplementary Table 1. ICP results of Pt-Ni NWs and Pt-Ni NWs-S.

ICP	Pt /At%	Ni /At%
Pt_3Ni_1 NWs	74.4	25.6
Pt_3Ni_2 NWs	59.1	40.9
Pt_3Ni_3 NWs	48.8	51.2
Pt_3Ni_4 NWs	41.6	58.4
Pt_3Ni_1 NWs-S	77.6	22.4
Pt_3Ni_2 NWs-S	60.9	39.1
Pt_3Ni_3 NWs-S	50.9	49.1
Pt_3Ni_4 NWs-S	42.7	57.3

Supplementary Table 2. Summary of HER properties of the high performance electrode materials reported in recent literatures. (The current densities in the table are normalized to the geometric area of electrode).

**Sample**	**Loading (mg cm^{-2})**	**Overpotential at 10 mA cm^{-2} (mV)**		**Current Density at -0.07 V (mA cm^{-2})**		**Reference**
		**0.1M KOH**	**1M KOH**	**0.1M KOH**	**1M KOH**	
$Ni(OH)_2$/ Pt-islands/ Pt(111) surface	——	~138	——	~2.2	——	*Science* **2011**, *334*, 1256
$Ni(OH)_2$/ Pt/C (TTK)	0.06	~90	——	~9	——	*Science* **2011**, *334*, 1256
Pt NWs/SL - $Ni(OH)_2$	0.016	~48	~70	26.6	10.9	*Nat. Commun.* **2015**, *6*, 6430
$Ni(OH)_2$ modified Pt surface	——	~75	——	~9.5	——	*Angew Chem. Int. Ed.* **2012**, *124*, 12663
$Ni(OH)_2$ modified Ir surface	——	~64	——	~15	——	*Angew Chem. Int. Ed.* **2012**, *124*, 12663
$Zn_{0.30}Co_{2.70}S_4$ nanocrystals	0.285	——	85	——	~7	*J. Am. Chem.* Soc. **2016**, *138*, 1359
$Ni_{0.33}Co_{0.67}S_2$ NWs /Ti foil	0.3	——	88	——	~7	*Adv. Energy Mater.* **2015**, *5*,1402031
NiS nanoframes	2	——	~94	——	~7	*Angew Chem. Int. Ed.* **2015**, *54*, 5331
Ni_3S_2/Ni foam	1.6	——	223	——	~1.5	*J. Am. Chem. Soc.* **2015**, *137*,

						14023
Ni_5P_4/Ni films	——	——	~150	——	~0	*Angew Chem. Int. Ed.* **2015**, *127,* 12538
NiO/Ni -CNT	0.28	——	~80	——	~8.5	*Nat. Commun.* **2014**, *5*, 4695
CeO_2/Ni -CNT	0.14	——	~91	——	~7.5	*Nano Lett.* **2015**, *15*, 7704
CoP films	2.71	——	~94	——	~5	*Angew Chem. Int. Ed.* **2012**, *54*, 6251
CoP/Carbon Cloth	0.92	——	~250	——	~0	*J. Am. Chem. Soc.* **2014**, *136*, 7587
MoP	0.86	——	~140	——	~0	*Energy Environ. Sci.* **2014**, *7*, 2624
Mo_2C@NC	0.28	——	~60	——	~12	*Angew. Chem. Int. Ed.* **2015**, *54*, 10752
MoCx	0.8	——	~151	——	~0	*Nat. Commun.* **2015**, *6*, 6512
**Pt_3Ni_2-NWs -S/C**	**0.015**	**45**	**42**	**20.2**	**37.2**	**This Work**
